# Exon expression in lymphoblastoid cell lines from subjects with schizophrenia before and after glucose deprivation

**DOI:** 10.1186/1755-8794-2-62

**Published:** 2009-09-22

**Authors:** Maureen V Martin, Brandi Rollins, P Adolfo Sequeira, Andrea Mesén, William Byerley, Richard Stein, Emily A Moon, Huda Akil, Edward G Jones, Stanley J Watson, Jack Barchas, Lynn E DeLisi, Richard M Myers, Alan Schatzberg, William E Bunney, Marquis P Vawter

**Affiliations:** 1 Department of Psychiatry and Human Behavior, Univ. of California, Irvine, CA, USA; 2 Psychiatric Genetics Research Center, Heredia, Costa Rica; 3 Hospital Nacional Psiquiatrico, Pavas, San Jose, Costa Rica; 4 Psychiatry, Univ. of California, San Francisco, CA, USA; 5 Molecular & Behavioral Neuroscience Institute, Univ. of Michigan, MI, USA; 6 Neuroscience Center, Univ. of California Davis, CA, USA; 7 Psychiatry, Cornell Univ., New York NY, USA; 8 Psychiatry, New York Univ., New York NY, USA; 9 Hudson Alpha, Huntsville AB, USA; 10 Psychiatry, Stanford University Palo Alto, CA, USA

## Abstract

**Background:**

The purpose of this study was to examine the effects of glucose reduction stress on lymphoblastic cell line (LCL) gene expression in subjects with schizophrenia compared to non-psychotic relatives.

**Methods:**

LCLs were grown under two glucose conditions to measure the effects of glucose reduction stress on exon expression in subjects with schizophrenia compared to unaffected family member controls. A second aim of this project was to identify cis-regulated transcripts associated with diagnosis.

**Results:**

There were a total of 122 transcripts with significant diagnosis by probeset interaction effects and 328 transcripts with glucose deprivation by probeset interaction probeset effects after corrections for multiple comparisons. There were 8 transcripts with expression significantly affected by the interaction between diagnosis and glucose deprivation and probeset after correction for multiple comparisons. The overall validation rate by qPCR of 13 diagnosis effect genes identified through microarray was 62%, and all genes tested by qPCR showed concordant up- or down-regulation by qPCR and microarray. We assessed brain gene expression of five genes found to be altered by diagnosis and glucose deprivation in LCLs and found a significant decrease in expression of one gene, glutaminase, in the dorsolateral prefrontal cortex (DLPFC). One SNP with previously identified regulation by a 3' UTR SNP was found to influence IRF5 expression in both brain and lymphocytes. The relationship between the 3' UTR rs10954213 genotype and IRF5 expression was significant in LCLs (p = 0.0001), DLPFC (p = 0.007), and anterior cingulate cortex (p = 0.002).

**Conclusion:**

Experimental manipulation of cells lines from subjects with schizophrenia may be a useful approach to explore stress related gene expression alterations in schizophrenia and to identify SNP variants associated with gene expression.

## Background

Microarray studies in postmortem brains of subjects with schizophrenia have implicated altered gene expression in pathways involved in myelination [1], GABAergic and glutamatergic transmission [2,3], synaptic plasticity [4,5] and metabolic pathways [6-8] [for review see [9]]. While examining gene expression in brain tissue from subjects with psychiatric or neurological disorders might be more relevant to disease pathology, assessment of whole blood or lymphocyte gene expression has several advantages, including practical availability of blood compared to brain tissue in human subjects and reduced confounds such as post-mortem interval (PMI), pH and agonal factors.

A number of studies have demonstrated the utility of using whole blood [10-12], transformed lymphocytes [3,6,13], peripheral blood mononuclear cells [14-17], and peripheral blood cells [18] as biomarkers in psychiatric and neurological disorders. These studies, while employing a variety of cell types, have been used to study panic disorder, nicotine dependence, post-traumatic stress disorder [15], schizophrenia [3,6,10,18], bipolar disorder [10], Huntington disease [11], epilepsy [12], Tourette syndrome [12], Down syndrome and Alzheimer's disease [13,16]. In addition to increased availability of lymphocytes and reduced confounds such as PMI, pH and agonal factors, transformed lymphocytes have the advantage of being more readily amenable to experimental manipulations than postmortem brain tissue. While it may be argued that state-dependent and brain tissue-dependent features are avoided by use of the cell lines, more stable genetic features may be prominent. Furthermore, previous work found a moderate correlation between blood and gene expression from 17 brain regions in humans, and reported that about half of a predetermined set of candidate genes relevant to schizophrenia were expressed in both whole blood and prefrontal cortex [19]. Experimental manipulation of cell lines from subjects with schizophrenia may be a useful approach to explore stress related gene expression alterations in schizophrenia and to identify SNP variants associated with gene expression. The purpose of this study was to examine the effects of glucose reduction stress on LCL gene expression in subjects with schizophrenia compared to non-psychotic relatives.

Recently, Naydenov et al (2007) [20] examined the effects of glucose deprivation in lymphocytes to study stress-induced changes in gene expression in bipolar disorder compared to controls. Naydenov et al (2007) [20] reported significant differences between subjects with bipolar disorder and controls in expression of genes related to the mitochondrial respiratory chain. This study demonstrated the advantages of combining a cellular model with experimental manipulation of glucose deprivation, which identified gene expression that was blunted in bipolar disorder but not controls.

The neural diathesis-stress model of schizophrenia proposes that in vulnerable individuals, psychosis can be triggered or worsened by stress and the interaction of stress with the dopamine system [21]. In addition to psychosocial stressors, metabolic stressors may also influence dopamine and stress responses. Previous work has also shown an increased dopamine response [22] and HPA activation [23] in subjects with schizophrenia compared to controls following metabolic stress due to 2-deoxyglucose treatment, although the molecular basis for this differential response is not understood. In the present study, it was hypothesized that a subset of genes would respond differentially to environmental manipulation (glucose reduction) in individuals with schizophrenia compared to controls.

Affymetrix 1.0 ST human exon arrays were utilized for this study to interrogate exon-level expression across the entire transcriptome. Exon arrays differ significantly from 3' expression arrays in the number and placement of the oligonucleotide probes and in the design of control probes for background correction. The Affymetrix 1.0 ST human exon array contains more than 5 million 25 bp oligomer probes, forming 1.4 million probe sets that together interrogate 1 million known and predicted exons up to four probes are selected from each putative exonic region. The coverage of the human 1.0 exon array represents over a 4-fold increase in probe density and an 8-fold increase in the number of perfect-match targets compared to the human U133 Plus 2.0 array. One advantage of the human 1.0 exon array platform is more robust measurements at the transcript level due to increased number of probes per transcript (30-40 probes across each entire RefSeq transcript compared to 11 probes mostly at the 3' transcript end in the U133 array). The probe sets are distributed along the entire length of the gene (as opposed to a concentration at the 3' end) allowing the potential to distinguish between different isoforms of a gene at the level of individual exons [24]. Therefore, this platform may identify alternative splicing events, which might play an important role in the pathophysiology of schizophrenia. Exon arrays have been demonstrated to attain improved sensitivity and specificity of absence/presence calls and may allow more accurate quantitative measurement of the level of gene expression compared to traditional 3' based arrays [25]. Quantitative real time PCR was used to validate exon array findings for 13 genes in lymphocyte samples. A subset of the validated genes was then assessed in brain samples from a separate cohort of subjects. Resequencing and TaqMan genotyping assays were conducted on one gene selected from exon array results in a larger cohort to identify gene variants associated with altered expression.

## Methods

### Subjects

Three cohorts were selected for this case-control study. Cohort 1 was used in the exon array analysis and consisted of LCLs from ten male subjects (five subjects with schizophrenia and five related non-psychotic subjects analyzed under normal and glucose deprivation conditions) for a total of 20 exon arrays (Table 1). The average age of controls and subjects with schizophrenia was 45 and 42, respectively. Cohort 2 was used for TaqMan genotyping assays on a total of 541 subjects (111 subjects with bipolar disorder, 19 subjects with major depressive disorder, 172 subjects with schizophrenia and 239 controls). Cohort 3 consisted of mRNA samples from two brain regions, dorsolateral prefrontal cortex (DLPFC) and anterior cingulate (AnCg), from 112 subjects, schizophrenia (n = 15), bipolar disorder (n = 17), major depressive disorder (n = 33) and controls (n = 47). Subjects with bipolar disorder and major depressive disorder were included in the analysis to test for the specificity of findings to schizophrenia. All three cohorts were obtained with IRB approval at the University of California, Irvine and cohorts 1 and 2 were obtained with IRB approval in Costa Rica. In cohort 3, 60 subjects were also genotyped (Table 2). Written, informed consent was obtained from all the individuals or from the surviving next-of-kin for all blood and brain samples reported in this study. Both cohorts 1 and 2 samples were collected from the Central Valley of Costa Rica in a relatively isolated population [26], and cohort 3 was collected in Southern California.

**Table 1:** Lymphoblastoid cell lines (LCLs) from ten subjects were grown under normal conditions or under glucose deprivation.

**Diagnosis **	**Relationship**	**Pair**	**Age**	**RIN** **(Normal Glucose)**	**RIN** **(Glucose Deprivation)**	**28S/18S ** **(Normal Glucose)**	**28S/18S** **(Glucose Deprivation)**
Schizophrenia	Brothers	1	43	9.8	10.0	2.0	1.7
Unaffected	Brothers	1	53	10.0	10.0	1.6	1.7
Schizophrenia	Brothers	2	48	9.5	10.0	1.6	1.9
Unaffected	Brothers	2	32	9.1	9.9	2.0	1.6
Schizophrenia	Brothers	3	45	9.6	9.6	1.6	1.5
Unaffected	Brothers	3	46	9.6	8.6	2.0	1.6
Schizophrenia	Uncle	4	33	10	10.0	1.9	1.7
Unaffected	Nephew	4	22	9.9	10.0	1.5	1.7
Schizophrenia	Cousins	5	47	9.4	10.0	1.7	1.5
Unaffected	Cousins	5	63	9.6	9.8	1.7	1.6

LCLs (N = 20) for five pairs of age- and sex- matched subjects before and after glucose deprivation were assayed by exon array. RNA quality was assessed by Agilent prior to exon array and all samples were found to have acceptable 28S/18S ratios and RNA Integrity Numbers (RIN).

**Table 2:** The dorsolateral prefrontal cortex (DLPFC) and anterior cingulate (AnCg) were dissected and RNA extracted from a total of 60 subjects.

**Diagnosis**	**N**	**DLPFC 28S/18S (Mean ± S.D.)**	**AnCg 28S/18S (Mean ± S.D.)**	**DLPFC RIN (Mean ± S.D.)**	**DLPFC RIN (Mean ± S.D.)**
BPD	7	2.26 ± 0.36	2.08 ± 0.38	7.87 ± 0.38	8.09 ± 0.65
Control	27	2.33 ± 0.46	2.11 ± 0.36	7.95 ± 0.31	8.04 ± 0.80
MDD	15	2.21 ± 0.32	2.02 ± 0.31	7.76 ± 0.53	8.01 ± 0.57
SZ	11	2.00 ± 0.43	1.90 ± 0.39	7.63 ± 0.80	8.36 ± 0.55

RNA quality was assessed prior to exon array and all samples were found to have acceptable 28S/18S ratios.

### Ficoll extraction and Epstein-Barr virus transformation

Approximately 10 mL of blood was centrifuged at 1500 rpm for 10 min at RT (25°C). The upper layer was transferred to a 15 ml Falcon tube containing 5 ml Ficoll-Paque Plus (GE Healthcare, Piscataway, NJ) and centrifuged for 20 min at 2500 rpm at RT. The buffy coat layer was transferred to a new tube containing 10 ml of 1.0 M PBS (pH = 7.4) and centrifuged at 1000 rpm for 10 min at RT. The pellet was resuspended in 400 μl of supernatant and an equal volume was added to two wells of a culture plate. One ml of concentrated Epstein-Barr virus stock was added to each plate well and the plate was incubated for 45 min at 5% CO_2 _and 37°C. Next, 10 μl of phytohaemagglutinin (PHA) was added to each well and 400 μl of transforming media RPMI 1640 containing 15% fetal bovine serum, 2 mM glutamine and 25 mg of gentamicin. The cell lines were grown until confluent and then stored frozen in RPMI supplemented media and 10% dimethylsulphoxide (DMSO) until being grown for the experiment.

### Glucose deprivation of lymphoblastoid cell lines

LCLs from cohort 1 subjects were grown under two environmental conditions (glucose deprived and normal glucose) to measure the effects of stress on exon expression and to examine state and trait-dependent alterations in exon expression in schizophrenia. All cell lines were thawed on the same date and grown with the same batch of RPMI-1640 supplemented media at 5% CO_2 _and 37°C to approximately the same cell density of 10^7 ^cells in a 25-cm^2 ^flask. The cells from the 25-cm^2 ^flask were split into two separate 75-cm^2 ^flasks and grown to a confluency of approximately 5×10^7 ^cells/flask. One flask from each subject was left to grow in normal glucose (2%) while the other flask of cells was transferred into complete media containing 5.55 mM d-glucose for 24 hours. These cells were then placed into low glucose media containing 2.77 mM d-glucose (0.5%) for 48 hours. Microscopic cell death was not apparent when cell counts were taken using trypan blue stain.

### Brain and lymphoblastoid cell line RNA extraction

Coronal slices of the brain were rapidly frozen on pre-cooled (to -120°C) aluminum plates, and stored at -80°C. The DLPFC and AnCg were later dissected and total RNA was extracted separately from each sample with TRIzol Reagent (Invitrogen, Carlsbad, CA) according to the supplier's protocol. Briefly, 1 ml of TRIzol was added to 5-10×10^6 ^cell pellets [10 ml of TRIzol per 50 ml confluent T75 flask of cells (pelleted)] or 50 mg DLPFC tissue. All final RNA pellets were resuspended in 100 μl of diethyl pyrocarbonate (DEPC) treated water. RNA degradation was assessed by quantifying the resulting 28S and 18S ribosomal band peak height ratios using the Agilent Bioanalyzer 2100 (Agilent, Palo Alto, CA) (see Tables 1 and 2).

### Affymetrix Human Exon 1.0 ST Array

The Affymetrix Human Gene Chip Exon 1.0 ST Array interrogates over one million exons representing over 17,868 NCBI Reference Sequence (RefSeq) transcripts. Arrays were run using the manufacturer's technical protocol (Affymetrix, Santa Clara, CA). Briefly, 2 μg total RNA was subjected to a ribosomal RNA removal procedure (RiboMinus Human/Mouse Transcriptome Isolation Kit, Invitrogen) to reduce the 28S and 18S rRNA population in order to minimize background and increase sensitivity of the assay. Reduced RNA was then reverse-transcribed to cDNA using random hexamers tagged with a T7 promoter sequence followed by a second strand cDNA synthesis using DNA polymerase (GeneChip WT cDNA Synthesis Kit, Affymetrix). The resulting double stranded cDNA was then used for amplification of antisense cRNA and cleaned using the Gene Chip Sample Cleanup Module (Affymetrix). A second cycle cDNA synthesis was then performed using random primers to reverse transcribe the cRNA into sense single stranded DNA which was then fragmented, labeled, and hybridized to Affymetrix Human Gene Chip Exon 1.0 ST Arrays. Arrays were washed, stained, and scanned on the Affymetrix Fluidics Station and G7 Affymetrix high-resolution scanner using GCOS 1.3. Gene expression traits were derived from the cel files and analyzed with a Robust multi-array average (RMA) procedure [27].

### Quantitative polymerase chain reaction (qPCR) of candidate diagnosis genes

The 100 μl reverse transcription reaction consisted of RNase-free water, TaqMan reverse transcriptase buffer (10 μl), 25 mM MgCl_2 _(22 μl), dNTPs (20 μl), Oligo d(T)_16 _(5 μl), RNase inhibitor (2 μl), MultiScribe Reverse Transcriptase, and 2 μg RNA from brain or lymphocytes. Real-time PCR was performed using the ABI Prism 7000 Sequence Detection System^® ^(Applied Biosystems™), using the default thermal cycler program for all genes: 10 min of pre-incubation at 95°C followed by 40 cycles for 15 sec at 95°C and 1 min at 60°C. Individual real-time PCR reactions were carried out in a volume of 25 μl in 96-well plates (Applied Biosystems™) containing 12.5 μl SYBR Green, 5 μl cDNA (4 ng/μl), and 7.5 μl H_2_O. At the end of each reaction, the cycle threshold (Ct) was manually set at the level that reflected the best kinetic PCR parameters. Although each RNA sample was either DNase treated or Qiagen column purified, to further increase gene specificity one primer in each pair was designed (Primer Express, ABI) to span two exons and primers were not 3' UTR biased. The primers were tested for amplification of any residual genomic DNA contamination. The dissociation curves of real time PCR were monitored for primer-dimer pairings, which interfere with SybrGreen fluorescence measurements. A melting curve analysis was used for all primers and any primers that displayed amplification of genomic DNA less than 35 Ct were discarded and redesigned. (See Additional File 1 for primer sequences.)

### TaqMan SNP genotyping

DNA samples were genotyped for an IRF5 variant (rs10954213) using TaqMan 5'-allele discrimination assays (Applied Biosystems, Foster City, CA). Allele-specific probes were labeled with the fluorescent dyes VIC and FAM, respectively. The PCR reaction was carried out in a total reaction of 5 μl (1 μl DNA, 2.5 μl TaqMan master mix, 0.125 μl 40× a IRF5 assay, 1.375 μl H_2_O) using the following amplification protocol: denaturation at 95° for 10 minutes, followed by 50 cycles at 92° for 15 seconds and at 58°C for 1.5 minutes. The genotype of each sample was determined by measuring allelic-specific fluorescence using the automated ABI Prism 7900 Sequence Detection system, with SDS 2.3 software for allelic discrimination (Applied Biosystems). Duplicate samples and negative controls were included to check the accuracy of genotyping.

### Identification of SNPs associated with candidate gene expression

A second aim of this project was to identify cis-regulated transcripts associated with diagnosis. The *mRNA by SNP Browser v 1.0.1 *software was used to identify SNPs within the interferon regulatory factor 5 (IRF5) that were strongly related to expression and the regions around these SNPs were sequenced in subjects with exon array data. The *mRNA by SNP Browser *software [28-30] contains association results of 54,675 transcripts with 406,912 SNPs (p < 0.05) and allows SNPs to be visualized in their genomic context along with linkage disequilibrium maps and putative haplotype blocks derived from the analysis of over 3 million SNPs genotyped in several populations by the International HapMap project. The IRF5 SNP with the most significant p-value related to expression in cohort 1 was followed up to examine whether there was an association between genotype and diagnosis in cohort 2, and genotype and expression in cohort 3.

### Data analysis

Data were analyzed in Partek Genomic Solutions (St. Louis, MO) by a repeated measure ANOVA (mixed model) with Diagnosis × Probeset, Glucose deprivation × Probeset, Diagnosis × Probeset as main effects and corresponding interactions. Transcript expression was estimated by an average of the expression levels across all probesets in each RefSeq transcript. Probeset effects were examined to identify exon specific effects of diagnosis or glucose deprivation. The threshold for significance was obtained using a Benjamini-Hochberg step-down FDR cutoff of 25%.

### Ingenuity pathway analysis

Genes with a step-down FDR less than or equal to 25% were used as the input variables for the data set to query the Ingenuity Pathways Analysis (IPA) software v7.1 canonical pathway analysis. Gene symbols were mapped to corresponding gene objects in the Ingenuity Pathways Knowledge Base. Each network or pathway was set to have a maximum of 35 focus genes and IPA identified those pathways that were most significant to the input data set. The significance of the association between the data set and the canonical pathway was determined based on the Benjamini-Hochberg step-down FDR calculated with the Fisher's exact test by calculating the probably that the association between the genes in the data set and the canonical pathway is due to chance alone.

## Results

**Figure 1:**
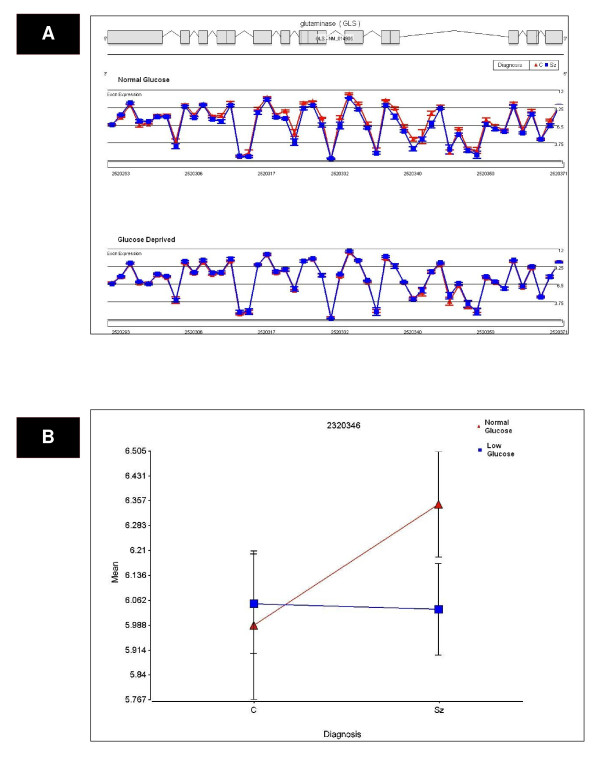
**(a) A significant Diagnosis × Glucose deprivation × Probeset interaction effect on GLS expression was detected by exon array**. Exon array probeset expression levels are shown for the entire transcript. The probe set number is shown on the x-axis and the average expression for each diagnosis group is depicted on the y-axis. There was a significant decrease in probeset expression in subjects with schizophrenia (shown in blue) compared to controls (shown in red), under normal glucose conditions (b) The probeset with the most significant Diagnosis × Glucose interaction was probeset 2520346 (p = 0.001). Posthoc analysis revealed a significant decrease in GLS probeset expression in subjects with schizophrenia before and after glucose deprivation compared to normal controls (p = 0.001 and 0.0002, respectively).

**Figure 2:**
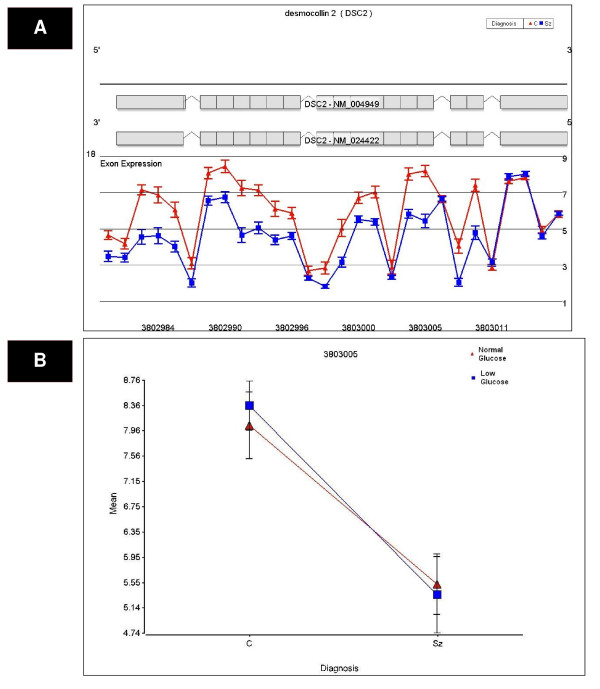
**A significant Diagnosis × Glucose deprivation × Probeset interaction effect on DSC2 expression was detected by exon array**. (a) Exon array probeset expression levels are shown for the entire transcript. The probe set number is shown on the x-axis and the average expression for each diagnosis group is depicted on the y-axis. The probe set number is shown on the x-axis, and the average expression for each diagnosis group is depicted on the y-axis. There was a significant Diagnosis × Probeset interaction effect on DSC2 expression, with a significant decrease in DSC2 expression in subjects with schizophrenia (shown in blue) compared to controls (shown in red). (b) The DSC2 probeset with the most significant diagnosis effect was probeset 3803005 (p = 2.06 × 10^-5^) and is depicted by dot plot. These findings were validated by qPCR (p = 0.0038).

**Figure 3:**
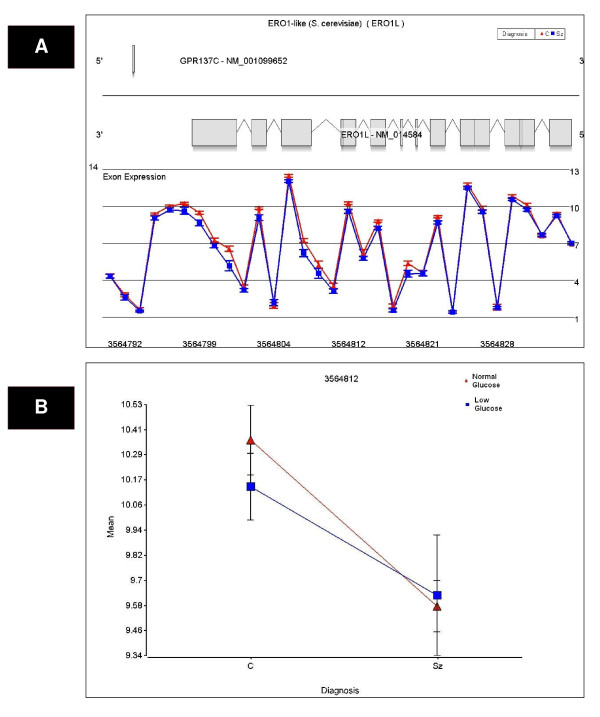
**A significant Diagnosis × Glucose deprivation × Probeset interaction effect on ERO1L expression was detected by exon array**. (a) Exon array probeset expression levels are shown for the entire transcript. The probe set number is shown on the x-axis and the average expression for each diagnosis group is depicted on the y-axis. There was a significant Diagnosis × Probeset interaction effect on ERO1L expression, with a significant decrease in ERO1L expression in subjects with schizophrenia (shown in blue) compared to controls (shown in red). (b) The probeset with the most significant diagnosis effect was probeset 3564812 (p = 0.0025) and is depicted by dot plot. These findings were validated by qPCR (p = 0.0037).

We tested the effects of glucose deprivation on LCL transcript profiles in subjects with schizophrenia compared to non-affected relatives. Diagnosis and glucose deprivation by probeset interaction effects were examined to identify exon-specific changes in expression. Transcripts were identified with significantly altered expression using an FDR cut-off of 25%. This cut-off was chosen based upon previous work by Benes et al, 2006 [31], which suggests an FDR cutoff of 5% may be too stringent for pathway analyses. There were no overall effects of diagnosis using a step-down FDR (Benjamini-Hochberg) cutoff of 25%. Step-down FDR is more conservative than step-up FDR and gives similar numbers of findings compared to Bonferroni correction [32]. Using a Benjamini-Hochberg step-down FDR cutoff of 25% there were 122 significant Diagnosis × Probeset effects, 328 Glucose deprivation × Probeset effects and 8 Diagnosis × Glucose deprivation × Probeset effects (see Additional file 2 for further detail; see Figures 1, 2 and 3 and Additional file 3 for exon array expression levels of representative genes GLS, DSC2 and ERO1L). Bonferroni correction of the mixed model ANOVA results was initially considered, but this approach assumed independent relationship among transcripts and these assumptions would be violated. Nevertheless, with a Bonferroni correction of 5%, no overall diagnosis effects, 72 Diagnosis × Probeset effects, 222 Glucose deprivation × Probesets effects and 5 Diagnosis × Glucose deprivation × Probeset effects were significant.

### QPCR validation of top ranked genes in cohort 1

Thirteen candidate genes were chosen for qPCR validation based on p-value and fold-change of probesets with altered expression between schizophrenic and non-psychotic relatives' cell lines. Eight of 13 schizophrenia candidate genes were validated by qPCR (Table 3) for an overall validation rate by qPCR of 62% (p < 0.05, two tailed t-test) while the remaining 5 genes showed a trend for validation by qPCR (p < 0.1, two tailed t-test). All qPCR results were concordant compared to microarray in terms of the direction of fold changes. The exon array expression of three genes validated by qPCR (GLS, ERO1L and DSC2) is shown in Figures 1, 2 and 3).

**Table 3:** QPCR of LCLs was performed to confirm altered gene expression in twelve top schizophrenia candidate genes identified by exon array analysis from Diagnosis × Probeset interactions.

**Gene** **Symbol**	**Diagnosis (p-value)**	**Glucose (p-value)**	**Diagnosis × Glucose (p-value) **	**Mean ΔCt (C-GD)**	**Mean** **ΔCt (C-NG)**	**Mean ΔCt (SZ-GD)**	**Mean ΔCt** **(SZ-NG) **	**FC**
**ERO1L**	**0.0037**	**0.6354**	**0.998**	**-1.45**	**-1.31**	**-0.41**	**-0.26**	**0.48**
**DSC2**	**0.0038**	**0.5318**	**0.999**	**0.18**	**0.64**	**2.58**	**3.03**	**0.19**
**MCCC2**	**0.0057**	**0.0006**	**0.713**	**-2.23**	**-1.31**	**-1.53**	**-0.76**	**0.65**
**CR1**	**0.0068**	**0.4561**	**0.797**	**0.72**	**1.13**	**2.06**	**2.26**	**0.42**
**IRF5**	**0.0104**	**0.9943**	**0.897**	**-0.27**	**-0.21**	**-1.63**	**-1.69**	**2.68**
**GLS**	**0.0325**	**0.9193**	**0.441**	**0.515**	**0.234**	**-0.756**	**-0.380**	**1.93**
**DSC3**	**0.0458**	**0.5623**	**0.709**	**4.38**	**4.62**	**6.42**	**7.53**	**0.18**
**ADCY1**	**0.0478**	**0.0083**	**0.893**	**2.34**	**3.97**	**3.52**	**5.00**	**0.46**
PPFIBP1	0.0630	0.1272	0.746	1.83	2.54	2.69	3.16	0.60
PDE4D	0.0788	0.2563	0.691	2.81	3.66	4.03	4.44	0.50
ZC3HAV1	0.0975	0.9714	0.922	-0.07	-0.03	1.18	1.09	0.44
TNIK	0.1037	0.0390	0.950	0.86	2.21	1.90	3.18	0.50
HEBP2	0.1075	0.7067	0.976	-0.07	0.19	1.15	1.45	0.42

These genes were selected for validation after examining probeset-specific expression for diagnosis related differences. The ΔCt was calculated by subtracting the Ct of the gene of interest from the Ct of the housekeeping gene SLC9A1. Fold change was calculated as 2^-(schizophrenia mean -- control mean). (C -- Control; SZ -- Schizophrenia; GD -- Glucose deprivation; NG -- Normal glucose; FC -- Fold change). The direction of fold change between diagnosis groups using microarray and qPCR data was consistent for 100% of the genes. A total of 62% of attempted validations were significant when gene expression was measured by qPCR. Bold data was significant by microarray and qPCR.

### Ingenuity pathway analysis

**Figure 4:**
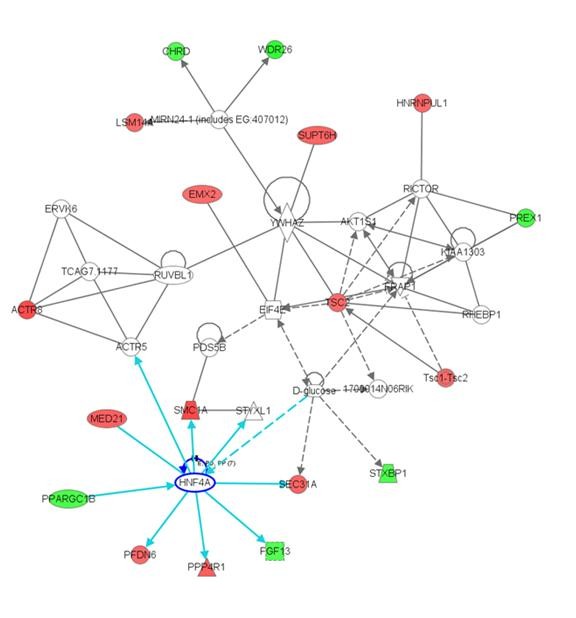
**IPA analysis of genes with Diagnosis × Glucose deprivation × Probeset effects identified a network of 18 genes**. Red indicates genes upregulated in schizophrenia in the glucose-deprived condition, green indicates genes down-regulated in schizophrenia in the glucose deprived condition, compared to normal controls in the glucose normal condition. White indicates the genes were not part of the dataset file genes. Solid lines indicate direct interactions, while dashed lines indicate indirect interactions. The network shapes are indicative of the molecular class (oval -- transcription regulator; square -- cytokine, trapezoid -- transporter; diamond -- enzyme; double circle -- complex/group; circle -- other). The network included 18 genes that showed diagnosis-dependent expression alterations following glucose deprivation, with Hepatocyte nuclear factor 4, alpha (HNF4A) as one node in this gene network.

**Figure 5:**
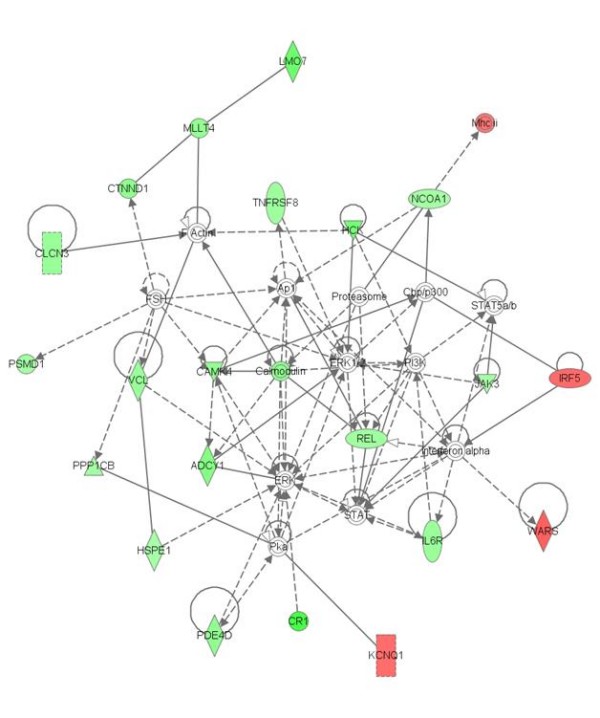
**IPA analysis of genes with Diagnosis × Probeset effects identified a network of 22 genes**. Red indicates genes upregulated in schizophrenia, green indicates genes down-regulated in schizophrenia. White indicates the genes were not part of the dataset file genes. Solid lines indicate direct interactions, while dashed lines indicate indirect interactions. See Figure 4 legend for description of molecular classes indicated by each network shape. The network included 22 genes that showed diagnosis effects on gene expression. Interestingly, calcium-activated calmodulin, which has previously been shown to be altered in expression in schizophrenia [49], was a centrally located complex within this network.

Ingenuity pathway analysis was conducted using all transcripts with p-values below a Benjamini-Hochberg step-down FDR cutoff of 25%. For the 8 Diagnosis × Glucose deprivation × Probeset effect genes, IPA identified a network involving the transcription factor HFN4A, which was associated with disease specific responses to glucose deprivation. To increase the number of genes for an exploratory analysis, we examined a larger set of 67 Diagnosis × Glucose deprivation × Probeset effect genes identified by *step-up *FDR cutoff of 25% and a larger network involving transcription factor HFN4A was identified (see Figure 4). The three most significant functional categories were molecular transport, protein trafficking, and cellular function and maintenance (p < 0.05). The 122 genes with significant Diagnosis × Probeset effects based on a Benjamini-Hochberg step-down FDR cutoff of 25% were associated with no significant over-represented functional categories. A gene network of significant diagnosis effect genes is shown in Figure 5. The network includes 22 genes that showed diagnosis effects on gene expression. Calmodulin was a central complex located within this network. There were no over-represented functional categories for the 328 genes with significant Glucose deprivation × Probeset effects based on a Benjamini-Hochberg step-down FDR cutoff of 25%. There was not an over-representation of diagnosis-related genes implicated in schizophrenia.

### Resequencing to identify variants associated with altered gene expression in schizophrenia

A second aim of this study was to identify cis-regulated transcripts that were altered in schizophrenia to investigate gene variants that might be associated with genetic risk for schizophrenia. For all genes validated by qPCR, SNPs associated with expression were identified using the *mRNA by SNP Browser *[28-30]. SNPs with highly significant LOD scores associated with expression of IRF5 were identified within the IRF5 gene using the *mRNA by SNP Browser*, but not for other Diagnosis × Probeset effect genes that were validated by qPCR (Table 4). Regions (~300 bps) around SNPs of interest [33] were resequenced in 10 subjects from cohort 1. Two SNPs of interest (rs4728142 and rs10488630) adjacent to exon array probeset 3023264 showed a significant association between genotypes (G and A alleles, respectively) and increased microarray expression of IRF5 (Table 5). These SNPs showed high linkage disequilibrium. Three additional SNPs not listed in the *mRNA by SNP Browser *software (rs11770589, rs10954214 and rs10954213) were identified within IRF5 probe set 3023264 by re-sequencing. These SNPs were included in our analysis because of their proximity to SNPs shown to be associated IRF5 expression. There was a significant effect of genotype of SNPs rs4728142, rs10954214 and rs10954213 on IRF5 expression in cohort 1 LCLs (Table 5).

**Table 4:** There was a significant effect of genotype at two SNPs on IRF5 expression according to the *mRNA by SNP Browser *data which supported the investigation of the effects of these SNPs on probeset expression in the present study.

**Gene**	**Location**	**Affymetrix Probeset ID**	**SNP Marker**	**Effect Size**	**LOD**	**p-value**
IRF5	7q32	205468_s_at	rs4728142	-0.521	9.527	3.50E-11

IRF5	7q32	205468_s_at	rs10488630	-0.381	4.937	1.90E-06

The top 25 Diagnosis × Probeset transcripts were examined in *mRNA by SNP Browser *software [28,30,29] to identify SNPs associated with candidate gene expression from LCLs. This table lists the SNP associated with each Affymetrix U133 probeset ID, with the associated effect size, logarithm of the odds (LOD) score and p-value that were obtained from *mRNA by SNP Browser *data.

**Table 5:** Candidate SNPs within probesets were genotyped by resequencing and tested for the effect of genotype on exon array expression obtained in LCLs.

**SNP**	**F-statistic**	**p-value**
**rs10954213**	**17.24**	**0.0001**

**rs10954214**	**22.13**	**0.0004**

**rs4728142**	**4.17**	**0.04**

rs11770589	1.21	0.32

rs10488630	0.56	0.47

There was a significant effect of IRF5 SNP genotype on microarray expression of the IRF5 probeset 3023264, for 3 of the 5 SNPS tested by ANOVA.

### SNP rs10954213 association with IRF5 expression in cohorts 2 and 3

TaqMan SNP genotyping was performed for IRF5 SNP rs10954213 on DNA extracted from lymphocyte from 541 individuals (cohort 2) and brain samples from 60 individuals (cohort 3). This SNP was selected because it was the most significantly associated with expression in our study and was also previously shown to be associated with expression [33]. Cohorts 2 and 3 included subjects with bipolar disorder and major depressive disorder in addition to subjects with schizophrenia and controls to investigate the specificity of an association of the genotype with schizophrenia.

The association of the rs10954213 SNP was also investigated in a larger sample of subjects (cohort 2, N = 541) with schizophrenia, bipolar disorder and major depressive disorder. There was no significant association between diagnosis and this IRF5 SNP (chi-square = 0.26, p = 0.99). (See tables 6 and 7 for genotype frequencies within each cohorts 2 and 3). The genotype frequencies for each diagnosis group were in Hardy-Weinberg equilibrium.

**Table 6:** IRF5 rs10954213 allele and genotype counts are shown for Cohort 2.

**Rs10954213**	**Allele**		**Genotype**		
**Group**	**A**	**G**	**AA**	**AG**	**GG**

BPD	129	153	33	63	45

C	183	197	44	95	51

MDD	8	6	1	6	0

SZ	100	96	25	50	23

There was no significant relationship between allele and diagnosis group in cohort 2. The χ^2 ^statistic for each diagnosis group frequency comparison to control group frequency were as follows: BPD -- 0.37, p = 0.53; MDD -- 0.42, p = 0.51; SZ -- 0.42; p = 0.51.

**Table 7:** IRF5 rs10954213 genotype and allele counts are shown for Cohort 3.

**rs10954213**	**Allele**		**Genotype**		
**Group**	**A**	**G**	**AA**	**AG**	**GG**

BPD	11	7	4	3	2

C	43	31	11	21	5

MDD	20	10	7	6	2

SZ	14	14	3	8	3

There was no significant relationship between genotype frequency and diagnosis group in cohort 3. The Odds ratio (OR) and p-value statistic for each diagnosis group frequency comparison to control group frequency were as follows: BPD -- OR = 1.13, p = 1.0; MDD -- OR = 1.44, p = 0.50; SZ -- OR = 0.72, p = 0.51).

**Figure 6:**
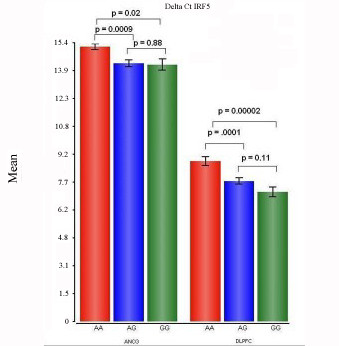
**The relationship between IRF5 expression (measured by qPCR of the 3'-UTR of IRF5 in two regions of brain in cohort 3) and the IRF5 SNP rs10954213 genotype is shown**. The ΔCt was calculated by subtracting from the Ct IRF5 the average Ct of two housekeeping genes (SNX16 and TSR1 in the anterior cingulate and SDHA and CAMK2A in the dorsolateral prefrontal cortex). A larger ΔCt indicates less expression of the target gene. The relationship between rs10954213 genotype and IRF5 probeset 3026432 expression was significant in these subjects in the DLPFC (F = 11.76; df = 1, 58;p = 8.11 × 10-5) and anterior cingulate cortex (F = 5.55; df = 1,58; p = 0.007) from cohort 2. The fold changes of expression in the anterior cingulate of subjects with the AG and GG genotypes compared to those with the AA genotypes were 0.52 and 0.50, respectively. The fold changes of expression in the anterior cingulate of subjects with the AG and GG genotypes compared to those with the AA genotypes were 0.33 and 0.38, respectively. Both regions showed a significant down-regulation in subjects with the AA genotype compared to the AG or GG genotype, consistent earlier reports of lower expression of the long 3'UTR in subjects carrying G alleles [33].

The expression of IRF5 was studied in cohort 3 in the DLPFC. The relationship between rs10954213 genotype and IRF5 expression was significant in these subjects in the DLPFC (F = 11.76; df = 2, 58; p = 8.11 × 10^-5^) and anterior cingulate cortex (F = 5.55; df = 2, 58; p = 0.007) from cohort 3 (see Figure 6). However, there were no significant associations between diagnosis and IRF5 expression in the DLPFC (F = 0.04; df = 1,59; p = 0.996) or the anterior cingulate (F = 0.28; df = 1,59; p = 0.84), nor Genotype × Diagnosis interaction effect on expression in the DLPFC (F = 0.90; df = 5, 55; p = 0.52) or the anterior cingulate (F = 0.90; df = 5, 55; p = 0.84).

### Brain gene expression of candidate biomarkers

Five additional genes with significantly altered probeset expression in LCLs between schizophrenia and unaffected relatives (ADCY1, DSC2, DSC3, GLS and PDE4D) were examined by qPCR expression in DLPFC tissue from subjects with schizophrenia and non-psychotic controls in cohort 3. The direction of change of GLS in both LCLs and the prefrontal cortex was consistent and decreased in schizophrenia in LCLs under glucose deprivation and the DLPFC. For comparison, subjects with bipolar disorder (BPD) and major depressive disorder (MDD) were included in the analysis. There was a significant decrease in expression of GLS in subjects with schizophrenia compared to controls (p = 0.04, Fold Change = 0.73), but no significant alterations of brain gene expression in schizophrenia of any other transcript examined. There were no significant alterations in brain expression of any of the other transcripts examined p-values for the in schizophrenia (see Table 8), although ADCY1 was down-regulated in major depressive disorder and bipolar disorder.

**Table 8:** The QPCR results showed a significant decrease in brain gene expression of GLS in subjects with schizophrenia compared to controls.

**Genes**	**BPD** **FC**	**MDD** **FC**	**SZ** **FC**	**BPD versus C p-value**	**MDD versus C p-value**	**SZ versus C p-value**
**PDE4D**	1.08	1.00	0.96	0.42	0.99	0.61

**GLS**	0.99	0.99	0.73	0.94	0.92	0.04

**DSC2**	0.99	1.06	0.97	0.93	0.40	0.66

**DSC3**	0.96	1.00	1.01	0.75	0.98	0.94

**ADCY1**	0.88	0.87	1.00	0.03	0.00	0.95

No significant alterations of DLPFC gene expression in schizophrenia of any other transcript examined. BPD -- bipolar disorder; C -- control; MDD --major depressive disorder; SZ -- schizophrenia; FC -- Fold Change

## Discussion

The results of this study suggest exon arrays in conjunction with cell lines and brain samples may be a valuable tool to identify gene expression associated with genotype in the context of psychiatric disorders. Despite a relatively small sample of cell lines grown under two different conditions, this study identified eight differentially expressed transcripts between cases and controls when compared under two glucose conditions, 328 transcripts with significant Glucose deprivation × Probeset effects on expression, and a total of 122 transcripts with significant Diagnosis × Probeset effects on expression demonstrating probeset-specific effects of diagnosis and glucose deprivation on expression. These transcripts passed a 25% step-down Benjamini Hochberg false discovery and the complete dataset is available by request.

There were no significant main effects of diagnosis. This might be due to the fact analyses were performed using summarized transcript data and gene transcript expression was estimated using the mean expression of all probesets, which for some transcripts could be 50 - 100 probesets. On the other hand, the Glucose deprivation × Probeset and Diagnosis × Probeset interaction effects were based on probeset data summarized at the exon level which was 4 - 8 probesets. The finding that expression differences within closely matched schizophrenia and unaffected relatives are restricted within certain exons of a transcript is consistent with the alternatively spliced transcripts being an over-represented significant category between schizophrenia and controls. As the exon array does not contain junction probes, this conclusion is speculative, as the exon array would measure both spliced and unspliced transcripts, and does not actually differentiate spliced and unspliced transcripts except in situations where splicing is an all or none occurrence. Over 70% of human multi-exon genes are alternatively spliced [34]. Also, limited power may have played a role in our lack of diagnosis or glucose deprivation effects. The effect sizes detected for 100 most significant diagnosis effects ranged from 0.05 to 1.19. A power analysis done in G*Power showed 80% power to detect an effect size of 0.55 [35].

There is a growing body of evidence of glucose metabolism abnormalities in schizophrenia. In a study by Ryan et al (2003), more than 15% of subjects with schizophrenia had impaired fasting glucose tolerance [36]. Subjects with schizophrenia also had higher fasting plasma levels of glucose, insulin and cortisol and were more insulin resistant than control subjects [36]. There is also evidence for linkage between enzymes that control glycolysis and schizophrenia, including PFKFB2, a rate limiting enzyme in glycolysis, and hexokinase, a regulatory enzyme in the metabolic pathway [37]. Insulin has been shown to increase the expression of genes that are decreased in the hippocampus in subjects with schizophrenia in neuroblastoma cells [9]. Moreover, glucose administration improves verbal declarative memory in clozapine-treated subjects with schizophrenia [38], although the mechanism of action remains unknown. Previous research has implicated metabolic and mitochondrial genes [3,7,8,39,40], including ornithine aminotransferase (OAT) [7] and metabolic enzyme cytosolic malate dehydrogenase (MDH1) [3,41] in schizophrenia, which have not been shown to be influenced by cigarette smoking or antipsychotic treatment. For these reasons, we examined the effects of glucose deprivation on gene expression in subjects with schizophrenia compared to controls.

Of the significantly altered transcripts identified in this study, only GLS showed significant Diagnosis × Probeset, significant Glucose deprivation × Probeset, and significant Diagnosis × Glucose effects. This gene and four other Diagnosis × Probeset effect genes were selected for further study in the DLPFC and only GLS was significant in DLPFC. Glutaminase, which converts glutamine to glutamate, was decreased in expression in glucose-deprived LCLs and decreased in expression in the DLPFC in subjects with schizophrenia compared to controls. This is in contrast to previous findings showing increases in glutaminase in the thalamus [42] and in DLPFC [43] of subjects with schizophrenia. In addition to GLS expression being regulated by cellular environment, the effects of diagnosis and glucose on expression varied by exon and may be isoform-specific. This gene may be worth further association study by SNP analysis or resequencing. In addition to glutaminase, there was a significant Diagnosis × Glucose deprivation × Probeset effect on PPARGC1B expression. PPARGC1B is located at 5q33, which has been previously linked to schizophrenia [44] and PPARGC1B participates in mitochondrial biogenesis and plays a role in fat oxidation and nonoxidative glucose metabolism [45], which is interesting in light of evidence of glucose metabolism abnormalities and mitochondrial dysfunction in schizophrenia. None of the other Diagnosis × Glucose deprivation × Probeset effect genes that passed a step-down FDR cutoff of 25% is known to play a role in glucose metabolism or to be sensitive to metabolic stressors. Thus, induction of gene expression changes in vitro by glucose reduction could be compared to increased glucose concentrations which may alter mitochondrial and metabolic function.

IRF5 expression was down-regulated in cohort 1. We examined the relationship between Interferon Regulatory Factor 5 (IRF5) expression and genotype and found expression of IRF5 to variation at SNP rs10954213 in all cohorts. However, IRF5 gene expression alteration in LCLs or brain was not observed in schizophrenia in a larger sample of subjects by RT-PCR and no SNP association with schizophrenia was observed.

IPA network analysis of transcripts with significant Diagnosis × Glucose deprivation × Probeset effects revealed a network of 18 genes that showed diagnosis-dependent expression alterations following glucose deprivation. D-glucose and hepatocyte nuclear factor 4, alpha (HNF4A) were two nodes in this gene network. HNF4A mutations have been associated with type II diabetes [46-48]. This gene is located at 20q12, a previously identified locus of schizophrenia [44]. This gene is interesting in light of findings of increased prevalence of diabetes in schizophrenia [28], and further investigation of HNF4A variants in schizophrenia may be warranted. IPA network analysis of transcripts with significant Diagnosis × Probeset effects revealed a network of 18 down-regulated and four up-regulated genes. Interestingly, calmodulin, which has previously been shown to be altered in expression in schizophrenia [49], was a centrally located complex within this network. If replicated, genes in these networks may represent novel targets for further study in schizophrenia.

The results of this study were compared with a recent study of microarray gene expression in the DLPFC of subjects with schizophrenia and control subjects [50]. Of the 54 genes identified by Maycox et al (2009) [50], there were 12 genes with diagnosis or Diagnosis × Probeset effects displaying nominal p-values < 0.05 in the current study (see Additional file 1). These results indicate that case and control derived cell lines might be a reliable and easily maintained resource for the study of the expression phenotype that is disease associated.

The human transcriptome was recently shown to have over 90% alternatively spliced variants [51] and it will be increasingly important to identify those variants at the exon level that differentiate cases and controls. We have shown that differential exon usage is associated with case - control differences, while few transcripts showed a complete differentiation across the entire gene; instead, differential expression was restricted to specific exons within a transcript. To fully pursue individual splice variants requires more validation experiments, but having a profile across the transcript represents a first approximation.

The use of exon arrays allowed for the possibility of detecting exon-specific changes in expression [24]. Previous work has shown a high level of agreement between digital counts by deep sequencing agreement with exon array [52]. In identifying gene expression alterations in LCLs, it may be important to combine theoretically valid experimental manipulations of cell lines from subjects with schizophrenia to induce gene expression alterations relevant to the disorder. An example would be to silence gene expression through siRNA transfection, and measure effects on known interactors.

### Expression of schizophrenia candidate genes in lymphoblastoid cell lines

One aspect of the present work was that many of the top candidate genes did not exhibit diagnosis or glucose deprivation effects. Although many schizophrenia candidate genes were expressed above background levels in LCLs, there was no overlap between the candidate genes identified in a recent meta-analysis of gene association studies [53] and genes with significant Diagnosis × Probeset × Glucose deprivation effects on expression found in this study. This may be due to tissue specificity (brain versus lymphocyte) of gene expression alterations in schizophrenia, or due to the selection of the control group, which consisted of relatives of subjects with schizophrenia and who likely carry many of the same genetic risk factors as their affected family members. Alternatively, reactive oxygen species generation might be increased more by increased glucose utilization [54], as we did not detect an over representation of mitochondrial or reactive oxygen genes by lowering glucose concentrations in cells. It is to be expected that increased glucose concentration would lead to more alterations in mitochondrial related genes.

A second aim of this study was to identify cis-regulated alterations in gene expression from the *mRNA by SNP Browser *[30] as one criterion for further study in the context of an association with the disease. There was a significant association between an IRF5 genotype and expression. A genotype effect of SNP rs10954213 on IRF5 expression was significant in LCLs, and was validated in the prefrontal cortex and anterior cingulate. However, there was no significant Diagnosis × Genotype effect on expression or a significant association of genotype with diagnosis in a larger sample for rs10954213. IRF5 exhibits genetically controlled polymorphic transcript variation [55]. The SNP rs10954213 variant in IRF5 is in the 3'- untranslated region (UTR) previously shown to influence IRF5 gene expression [33] in LCLs. This SNP creates a functional polyadenylation site and the A genotype correlates with increased expression of a transcript variant containing a shorter 3'-UTR [33], which was the same association found in LCLs, and brain tissue in the present study.

The 3'-UTR of mRNA has important functions in the stability, localization, and translation of mRNA [56]. MicroRNAs (miRNAs), are small noncoding RNAs that recruit an argonaute protein complex to a complementary target mRNA. Each miRNA-Ago complex interacts with a specific mRNA, typically through pairing of nucleotide bases between the miRNA sequence and complementary sequences in the mRNA's 3-untranslated region (3'UTR), which results in translation repression or degradation of the mRNA. A miRNA binding site for hsa-miR-185 was identified 1 bp downstream of SNP rs10954213 using the web based tool, MicroInspector [57]. It is possible that the effect of SNP rs10954213 variant on IRF5 protein levels may be mediated via this shortened 3'-UTR lacking this miRNA binding site. A further experiment to correlate reduction in protein expression with genotype would address this speculation.

We cannot rule out the possible effects of antipsychotics or cigarette smoking on gene expression. Recent research suggests that cigarette smoke and antipsychotic treatment may lead to gene-silencing effects through DNA methylation changes [58-60] which could lead to long-term changes in cell lines derived from smokers. The overlap between genes included in the IPA analysis and those known to be altered by antipsychotics or nicotine were assessed with Ingenuity and no overlap was observed.

## Conclusion

This study supports the use of experimental manipulation on LCLs to examine alterations in gene expression at the exon level in psychiatric disorders [3,61]. These results support the combined use of exon array, *mRNA by SNP Browser *software [30], and genotyping in the identification of gene variants that are associated with regulation of expression in cell lines to find disease specific associations.

## Competing interests

These authors (AM, WB, RS, HA, EGJ, SJW, JB, LED, RMM, AS, WEB, MPV) have received funding from Pritzker Neuropsychiatric Disorders Research Fund LLC for this work. RMM is a consultant for Bay City Capital and received reimbursement for his consulting work which was not related with the topic of this paper. All authors indicated (AM, WB, RS, HA, EGJ, SJW, JB, LED, RMM, AS, WEB, MPB) are applying for a patent related to the content of the manuscript (Patent Application 20080199866). The authors have no other competing interests.

## Authors' contributions

MVM performed computational analysis and generated qPCR, sequencing and TaqMan data. BR generated exon array data. PAS provided bioinformatics and statistical support. AM provided blood samples and diagnosis of living subjects. WB provided blood samples. RS provided diagnosis of postmortem subjects. EAM transformed and organized cell lines and assisted with genotyping. HA, EGJ, SJW and JB provided postmortem samples and critique of results. LED provided blood samples and diagnosis of living subjects. AS, RMM, WEB provided postmortem samples and critique of results. MPV contributed to the overall design of the project, provided critique of the results, participated in manuscript conceptual development and editing, and provided bioinformatics and statistical support. All authors read and approved the final manuscript.

## Pre-publication history

The pre-publication history for this paper can be accessed here:



## Supplementary Material

Additional File 1 **Primer sequences**. The tables lists primer sequences for all primers used in this study. Click here for file

Additional File 2 **significant diagnosis, glucose deprivation and probeset interaction effects on transcript expression overlap with previous findings in the prefrontal cortex of subjects with schizophrenia compared to controls**. Tables list transcripts with significant diagnosis × probeset, glucose deprivation × probeset and diagnosis × glucose deprivation × probeset effects, and overlap with previous findings in the prefrontal cortex of subjects with schizophrenia compared to controls. Click here for file

Additional File 3 **PPARGC1B and FBXW5 expression**. Figures displaying significant Diagnosis × Glucose deprivation × Probeset interaction effects on transcripts expression for genes PPARGC1B and FBXW5. Click here for file
