# Supplementary material for: Exon expression in lymphoblastoid cell lines from subjects with schizophrenia before and after glucose deprivation
Source: BMC Med Genomics. 2009 Sep 22;2:62. doi: 10.1186/1755-8794-2-62 (PMC2760574; doi:10.1186/1755-8794-2-62)
Supplement: Additional File 1 — Primer sequences. The tables lists primer sequences for all primers used in this study. [file 1755-8794-2-62-S1.DOC]

*Supplemental Table 1. Primer Sequences for qPCR*

DSC2 Forward: CTG ACC CTC GCG ATC TTAA TAT TT

DSC2 Reverse: GCA TCT AGT TTG GAG GGA ACA TG

DSC3 Forward: CCT GGC AAT AGA CAA AGA TGA TAG ATC

DSC3 Reverse: CTT GAA GTA TTT CTG GTG GAT TAT CAT TTT

ADCY1 Forward: CCT GCC AGG GAA GGT TCA TA

ADCY1 Reverse: CGT AAC CCG GTT CTA CCT CGT A

PDE4D Forward: GGT CAT TGA CAT CGT ACT TGC AA

PDE4D Reverse: TTA TCA AGA AGA AGA ACT CCA GAG CTT

ERO1L Forward: TGC TTC TGC CAG GGT AGT GGT TA

ERO1L Reverse: TGG GAA AAG CCT GTA GTT ATT AAA TCT

PPF1BP1 Forward: CTC CGT GGA CTT GGC AGA ATA T

PPF1BP1 Reverse: TCT AGA ACC ATG AGC CCA CCAT

TNIK Forward: GAC CTC ATC AAG AAC ACA AAA GGT AA

TNIK Reverse: TCA GCC CCC GTA AGA TTT CC

IRF5 Reverse: CTT CGA GAT CTT CTT CTG CTT TGG

IRF5 Forward: GCT ACA GGC ACC ACC TGT ACAG

HEBP2 Forward: CCC CAG CCC GGA AGT TA

HEBP2 Reverse: CCC GTC TGG ATG GCT GAAT

CR1 Forward: CAC ATG TGA AGT GAA ATC CTG TGA

CR1 Reverse: AGC TGG AGA TTT ACT GGA AAT AGCA

IRF5 Probe Set 3023264 FORWARD: 5’-ACT ACC AGT TGC TCC CAT GC

IRF5 Probe Set 3023264 REVERSE: 5’-ATT CCA CAC CCT TGC TTC AG

TNIK Forward: CGACACCCGCATGAGGAC

TNIK Forward: CGAATCAAAACGGGAATCCA

ERO1L Forward: TTA TCT AAG ATG GTT TCT GAG TGA A

ERO1L Reverse: CGC TAG TTT GAG AGA CTT AGA ACA

GLS Probe Set 2520346 Forward: TGT TTG GAT GAA ATT TGT G

GLS Probe Set 2520346 Reverse: CAA AAT GTA GGC ATA CTG GAA AA

*Supplemental Table 2 Primer Sequences for Sequencing*

Rs10488630 FORWARD: 5’-CCA ATA AAT CTG CAG GTC AGG

Rs10488630 REVERSE: 5’-AGGGAAGTGGTTCATTCTGC

Rs4728142 FORWARD: 5’- AGGCCATGTGAAGACAGAGC

Rs4728142 REVERSE: 5’-GCTTTGAAGTTTCTGGCACA

Rs10954214 and rs10954213 Forward: CCAAGAACCTGGAGCAGAAA

Rs10954214 and rs10954213 Reverse: CACCTCGGGGGACAATATAA
