# Supplementary material for: Exon expression in lymphoblastoid cell lines from subjects with schizophrenia before and after glucose deprivation
Source: BMC Med Genomics. 2009 Sep 22;2:62. doi: 10.1186/1755-8794-2-62 (PMC2760574; doi:10.1186/1755-8794-2-62)
Supplement: Additional File 2 — significant diagnosis, glucose deprivation and probeset interaction effects on transcript expression overlap with previous findings in the prefrontal cortex of subjects with schizophrenia compared to controls. Tables list transcripts with significant diagnosis × probeset, glucose deprivation × probeset and diagnosis × glucose deprivation × probeset effects, and overlap with previous findings in the prefrontal cortex of subjects with schizophrenia compared to controls. [file 1755-8794-2-62-S2.DOC]

*Supplemental Table 3.*

There were 122 genes with statistically significant Diagnosis x Probeset interaction effects on expression after Bonferroni correction. The gene symbol, mean expression level and p-value are shown in the table below. (C – Control; SZ – Schizophrenia; GD – Glucose Deprivation; NG – Normal Glucose; FC – Fold Change).

| **Gene Symbol** | **Mean (C+GD)** | **Mean (C+NG)** | **Mean**  **(SZ+GD)** | **Mean**  **(SZ+NG)** | **p-value (Diagnosis x Probeset)** |
| --- | --- | --- | --- | --- | --- |
| DSC2 | 6.36709 | 6.49745 | 4.80418 | 5.01118 | 1.07E-27 |
| ADCY1 | 6.48106 | 6.88675 | 5.83075 | 6.2725 | 1.67E-13 |
| JAK3 | 8.6024 | 8.57553 | 8.463 | 8.40987 | 1.46E-12 |
| EDNRA | 4.63083 | 4.78867 | 3.39967 | 3.4835 | 1.72E-11 |
| CENTD1 | 6.93805 | 6.92524 | 6.18065 | 6.65832 | 4.18E-11 |
| LOC440258 | 5.71628 | 5.72497 | 5.19179 | 5.24759 | 1.12E-10 |
| TP73L | 6.68017 | 7.05113 | 5.84765 | 6.1707 | 1.49E-10 |
| HEBP2 | 9.061 | 8.84175 | 8.62725 | 8.33975 | 1.54E-10 |
| ARG2 | 5.3374 | 5.13 | 4.8408 | 4.4654 | 2.10E-10 |
| AFF3 | 6.47917 | 7.133 | 5.59525 | 6.08275 | 2.50E-10 |
| DSC3 | 5.2353 | 5.5442 | 3.7057 | 4.0772 | 3.66E-10 |
| CLMN | 6.13663 | 6.47453 | 5.43432 | 5.52421 | 4.78E-10 |
| KIAA0870 | 7.58964 | 7.33145 | 6.79667 | 6.82836 | 6.99E-10 |
| XPO5 | 8.37589 | 8.4931 | 7.96658 | 8.36947 | 1.37E-09 |
| BBX | 7.64017 | 7.42687 | 7.02409 | 7.384 | 2.08E-09 |
| BAIAP2L1 | 7.0806 | 7.0972 | 6.2338 | 6.2816 | 2.18E-09 |
| C2orf28 | 7.92683 | 8.21587 | 7.683 | 8.12017 | 2.81E-09 |
| IL6R | 6.477 | 6.44456 | 6.10078 | 6.10033 | 3.03E-09 |
| MCCC2 | 8.63989 | 8.89922 | 7.96333 | 8.67811 | 3.48E-09 |
| SH2BP1 | 8.41662 | 8.71985 | 8.09062 | 8.55569 | 3.70E-09 |
| GLS | 8.82062 | 8.94821 | 8.31662 | 9.06 | 3.72E-09 |
| CTNND1 | 8.15469 | 8.17237 | 7.53506 | 7.77825 | 5.17E-09 |
| LGALS8 | 7.76063 | 7.70105 | 7.28379 | 7.39716 | 6.60E-09 |
| IRF5 | 8.40533 | 8.42367 | 8.44983 | 8.67033 | 1.41E-08 |
| PIP5K3 | 8.04838 | 7.96043 | 7.15281 | 7.576 | 1.57E-08 |
| ZC3HAV1 | 8.7933 | 8.88583 | 8.3027 | 8.77704 | 1.91E-08 |
| PODXL2 | 5.90583 | 5.99783 | 5.76033 | 5.79483 | 1.94E-08 |
| ZYG11A | 5.41167 | 5.76933 | 4.5 | 4.69278 | 1.98E-08 |
| WARS | 10.2578 | 10.1664 | 10.516 | 10.4042 | 4.29E-08 |
| IPO11 | 6.64419 | 7.05748 | 6.1211 | 7.04239 | 4.62E-08 |
| THADA | 6.64076 | 6.93293 | 6.17964 | 6.94511 | 5.34E-08 |
| ULK2 | 5.414 | 5.54097 | 4.76339 | 5.00309 | 6.49E-08 |
| SLC10A5 | 8.57057 | 8.64057 | 8.20829 | 8.47343 | 6.90E-08 |
| LNPEP | 10.0864 | 9.8636 | 9.35448 | 9.70432 | 9.68E-08 |
| AGPAT5 | 8.7003 | 8.7874 | 7.6872 | 8.48 | 1.02E-07 |
| HSPA4L | 6.5779 | 6.6268 | 5.9509 | 6.4594 | 1.05E-07 |
| PPT2 | 6.02014 | 6.0091 | 5.91703 | 5.80483 | 1.07E-07 |
| HIPK1 | 8.35209 | 8.11461 | 7.85565 | 8.06278 | 1.39E-07 |
| NCOA1 | 8.48106 | 8.72469 | 8.07556 | 8.725 | 1.40E-07 |
| SPATA6 | 3.386 | 3.35933 | 3.44307 | 3.30147 | 1.49E-07 |
| CLN5 | 5.99046 | 6.23938 | 5.94692 | 6.19754 | 1.64E-07 |
| FARP1 | 5.34514 | 5.3213 | 4.71978 | 4.56173 | 2.34E-07 |
| LMO7 | 6.44389 | 7.00274 | 5.4876 | 6.14103 | 2.49E-07 |
| MYO1D | 8.00761 | 8.29368 | 7.30574 | 7.69116 | 2.51E-07 |
| HSPE1 | 7.46867 | 7.65789 | 7.39678 | 7.42733 | 2.52E-07 |
| DKFZp762E1312 | 7.0376 | 7.57533 | 6.93453 | 7.39813 | 2.69E-07 |
| LY75 | 9.61723 | 9.38804 | 8.81604 | 9.06196 | 3.85E-07 |
| PPFIBP1 | 6.62933 | 6.67407 | 5.72874 | 6.16615 | 4.80E-07 |
| CXorf34 | 6.7527 | 7.1462 | 6.6485 | 6.9883 | 5.38E-07 |
| KDELC1 | 7.64706 | 7.92875 | 6.95106 | 7.71825 | 6.00E-07 |
| PSMD1 | 8.90007 | 9.14786 | 8.46321 | 9.14921 | 6.21E-07 |
| CR1 | 7.21453 | 7.24484 | 5.81516 | 6.18421 | 6.28E-07 |
| LRIG1 | 6.78672 | 7.13128 | 6.51744 | 6.76552 | 8.44E-07 |
| LOC440895 | 6.91533 | 7.19267 | 6.52667 | 7.04467 | 8.91E-07 |
| IGSF4 | 6.2135 | 6.6575 | 5.52867 | 5.6345 | 9.58E-07 |
| ARPC5L | 7.473 | 7.71575 | 7.39175 | 7.587 | 9.67E-07 |
| TNIK | 8.09833 | 8.51033 | 7.11144 | 7.93394 | 9.95E-07 |
| MAST2 | 7.13892 | 7.22232 | 6.85249 | 7.00692 | 1.03E-06 |
| TOP2B | 8.919 | 8.9701 | 7.88585 | 8.76895 | 1.08E-06 |
| ERO1L | 9.57329 | 9.45386 | 8.93043 | 8.94771 | 1.25E-06 |
| SAS10 | 7.37429 | 7.57 | 7.09486 | 7.64686 | 1.26E-06 |
| TNFRSF8 | 9.42916 | 9.192 | 9.07947 | 8.95189 | 1.28E-06 |
| PREX1 | 9.41698 | 9.31102 | 9.28451 | 9.31004 | 1.31E-06 |
| AER61 | 5.07661 | 5.43478 | 4.50922 | 5.1727 | 1.57E-06 |
| GARNL3 | 5.37724 | 5.66828 | 4.8369 | 5.00041 | 1.62E-06 |
| AQR | 7.66083 | 8.02694 | 7.23944 | 7.85872 | 1.93E-06 |
| SFRS7 | 8.63667 | 8.98947 | 8.25933 | 8.8476 | 2.10E-06 |
| MLLT4 | 6.70489 | 6.75696 | 5.98504 | 6.5577 | 2.48E-06 |
| TRIP | 6.00271 | 6.478 | 6.23314 | 6.39786 | 2.51E-06 |
| MARCH6 | 9.60626 | 9.44239 | 8.96768 | 9.40232 | 2.52E-06 |
| PDE4D | 5.46795 | 5.5065 | 4.8441 | 5.1215 | 2.71E-06 |
| ARHGAP6 | 5.9236 | 6.01384 | 5.41072 | 5.59232 | 2.73E-06 |
| PCMT1 | 8.32927 | 8.432 | 8.35491 | 8.66418 | 3.03E-06 |
| LRMP | 7.29064 | 7.70082 | 7.62391 | 7.935 | 3.24E-06 |
| PEX19 | 10.2169 | 10.2209 | 9.81255 | 10.2859 | 3.28E-06 |
| ALG5 | 6.637 | 7.02417 | 6.5695 | 7.0215 | 3.30E-06 |
| EML4 | 8.72133 | 8.8398 | 8.2332 | 8.66407 | 3.60E-06 |
| PKD2 | 6.5177 | 6.7141 | 5.9223 | 6.4013 | 3.72E-06 |
| SMARCA2 | 9.22819 | 9.59898 | 8.73256 | 9.30186 | 4.07E-06 |
| CLCN3 | 7.56356 | 7.62256 | 6.83811 | 7.56056 | 4.54E-06 |
| REL | 9.2485 | 9.18267 | 8.705 | 9.16867 | 4.61E-06 |
| EST1B | 7.85374 | 7.90774 | 7.62981 | 7.81039 | 4.82E-06 |
| C1orf2 | 7.48581 | 7.45457 | 7.46143 | 7.2361 | 5.01E-06 |
| LOC339843 | 7.46029 | 7.43814 | 6.89286 | 7.33271 | 5.07E-06 |
| ANKRD13C | 7.7873 | 7.90574 | 7.24261 | 7.82174 | 5.08E-06 |
| UBP1 | 7.93888 | 8.1956 | 7.6152 | 8.01304 | 5.16E-06 |
| IDS | 8.52538 | 8.453 | 8.4805 | 8.56775 | 5.25E-06 |
| FLJ42094 | 7.1385 | 7.26875 | 6.17592 | 6.44217 | 5.95E-06 |
| FLJ20485 | 6.14836 | 6.57327 | 5.37918 | 5.98636 | 6.06E-06 |
| SLC23A2 | 7.04157 | 7.0035 | 6.28371 | 6.22671 | 6.09E-06 |
| LAMP2 | 7.29433 | 7.51911 | 7.11944 | 7.45856 | 6.09E-06 |
| PPP1CB | 9.65267 | 9.57989 | 9.19033 | 9.58578 | 6.12E-06 |
| NR3C1 | 9.74 | 9.47313 | 9.20775 | 9.46087 | 6.14E-06 |
| CAMK4 | 9.20286 | 9.10486 | 8.57557 | 8.75871 | 6.42E-06 |
| SLC35F5 | 7.49038 | 7.4119 | 6.78257 | 7.35248 | 7.51E-06 |
| PGM2 | 8.3116 | 8.6084 | 7.69787 | 8.35453 | 7.70E-06 |
| ETFDH | 6.84863 | 7.03725 | 6.12087 | 6.8035 | 7.75E-06 |
| SLC4A7 | 6.7484 | 6.70543 | 5.82846 | 6.54183 | 7.83E-06 |
| CDC73 | 7.96457 | 8.04438 | 7.50029 | 8.00505 | 7.88E-06 |
| WDR36 | 8.48344 | 8.61416 | 7.77744 | 8.51152 | 8.02E-06 |
| ARHGAP18 | 7.0701 | 7.3567 | 6.2917 | 6.8307 | 8.34E-06 |
| BDP1 | 6.70021 | 6.74774 | 6.09779 | 6.70137 | 8.43E-06 |
| NDUFS1 | 8.53475 | 8.7615 | 7.98317 | 8.56992 | 8.55E-06 |
| HLA-DMA | 9.48356 | 9.77511 | 9.54689 | 9.75556 | 8.71E-06 |
| SCP2 | 7.58983 | 7.63496 | 7.13296 | 7.4187 | 8.82E-06 |
| SEC31L1 | 9.53339 | 9.5965 | 9.15417 | 9.66183 | 9.20E-06 |
| C1orf113 | 6.62825 | 6.12687 | 6.53963 | 5.96875 | 9.20E-06 |
| FLJ10808 | 7.71067 | 7.79426 | 6.89446 | 7.57138 | 9.66E-06 |
| VCL | 5.44081 | 5.64508 | 5.10205 | 5.24211 | 1.03E-05 |
| MGC3123 | 6.94267 | 7.116 | 6.9775 | 6.95317 | 1.07E-05 |
| HCK | 7.47738 | 7.26912 | 6.694 | 6.42875 | 1.18E-05 |
| NCOA7 | 7.04892 | 7.23277 | 6.25631 | 6.85615 | 1.19E-05 |
| MARLIN1 | 6.46011 | 6.20022 | 6.05022 | 5.98667 | 1.22E-05 |
| MGC52010 | 8.38333 | 8.48967 | 8.53967 | 8.479 | 1.22E-05 |
| GFM1 | 7.76408 | 7.98496 | 7.20296 | 7.8864 | 1.22E-05 |
| SEC15L1 | 5.46096 | 6.02226 | 5.06617 | 5.76887 | 1.23E-05 |
| FLJ20625 | 8.62667 | 8.95633 | 9.117 | 8.862 | 1.25E-05 |
| BCAT1 | 8.30484 | 8.14979 | 7.91 | 8.05695 | 1.32E-05 |
| PSTPIP2 | 7.18867 | 7.30433 | 6.56967 | 6.70817 | 1.32E-05 |
| LRPPRC | 9.4599 | 9.64443 | 8.57886 | 9.37257 | 1.37E-05 |
| GPR34 | 2.61829 | 3.082 | 3.23971 | 3.68971 | 1.39E-05 |

*Supplemental Table 4.*

There were 328 genes with statistically significant Glucose deprivation x Probeset interaction effects on expression after Bonferroni correction. The gene symbol, mean expression level and p-value are shown in the table below. (C – Control; SZ – Schizophrenia; GD – Glucose Deprivation; NG – Normal Glucose; FC – Fold Change).

| **Gene Symbol** | **Mean (C+GD)** | **Mean (C+NG)** | **Mean**  **(SZ+GD)** | **Mean**  **(SZ+NG)** | **p-value (Glucose x Probeset)** |
| --- | --- | --- | --- | --- | --- |
| KIF2C | 6.62658 | 7.17433 | 6.20617 | 6.93075 | 5.86E-15 |
| C2orf28 | 7.92683 | 8.21587 | 7.683 | 8.12017 | 1.86E-14 |
| GLS | 8.82062 | 8.94821 | 8.31662 | 9.06 | 8.03E-14 |
| SMC4L1 | 7.42211 | 7.95377 | 6.75131 | 7.72411 | 3.53E-12 |
| ILF3 | 8.73372 | 9.11489 | 8.63239 | 9.01883 | 7.30E-12 |
| ODF2 | 7.11888 | 7.35704 | 6.97808 | 7.24176 | 7.37E-12 |
| FARSLB | 9.08032 | 9.50053 | 8.71958 | 9.262 | 7.84E-12 |
| PFAS | 7.20238 | 7.55551 | 7.16703 | 7.41865 | 1.41E-11 |
| KDELC1 | 7.64706 | 7.92875 | 6.95106 | 7.71825 | 1.55E-11 |
| ATRN | 6.78766 | 7.07874 | 6.58657 | 7.0372 | 1.89E-11 |
| MCM4 | 8.2947 | 8.84113 | 8.06435 | 8.61148 | 2.11E-11 |
| SFXN2 | 6.84338 | 7.53846 | 6.78831 | 7.30308 | 3.61E-11 |
| ARID1A | 7.74489 | 7.97628 | 7.56856 | 7.9365 | 3.97E-11 |
| UBE2G1 | 9.09717 | 9.6195 | 9.17767 | 9.44517 | 5.73E-11 |
| ATP2C1 | 8.50044 | 8.67978 | 8.02033 | 8.68556 | 6.45E-11 |
| ASPM | 5.62405 | 6.35014 | 4.99781 | 6.08343 | 7.16E-11 |
| CDK5RAP2 | 6.73609 | 7.065 | 6.51213 | 6.97365 | 8.77E-11 |
| PTHLH | 4.46387 | 4.84525 | 4.71287 | 4.84487 | 8.94E-11 |
| RANBP5 | 9.52739 | 9.95103 | 9.16879 | 9.67133 | 9.43E-11 |
| TREX1 | 6.94661 | 6.94194 | 6.87782 | 6.85691 | 9.63E-11 |
| SEPT9 | 8.85345 | 8.81221 | 8.87772 | 8.70793 | 1.02E-10 |
| TMEM1 | 7.85568 | 8.13903 | 7.72981 | 8.03542 | 1.04E-10 |
| STAU2 | 6.08017 | 6.52748 | 5.86722 | 6.26609 | 1.05E-10 |
| AUH | 5.91493 | 6.11733 | 5.96213 | 6.046 | 1.29E-10 |
| CGI-09 | 7.618 | 8.08243 | 7.66857 | 7.97929 | 2.80E-10 |
| CHD3 | 7.55295 | 7.82777 | 7.29614 | 7.7045 | 2.83E-10 |
| COQ2 | 6.67938 | 7.37646 | 6.95877 | 7.44585 | 2.94E-10 |
| MYB | 7.786 | 8.77262 | 7.28625 | 8.36588 | 3.59E-10 |
| RERE | 7.53237 | 7.56611 | 7.47942 | 7.65774 | 3.79E-10 |
| AKAP8 | 7.8107 | 8.0398 | 7.6896 | 8.0871 | 4.10E-10 |
| KIAA0690 | 6.62168 | 7.02489 | 6.62221 | 7.002 | 4.11E-10 |
| KIAA2010 | 7.14465 | 7.41788 | 6.80088 | 7.367 | 4.77E-10 |
| TRPM7 | 8.02041 | 8.26773 | 7.40605 | 8.00609 | 4.87E-10 |
| COL19A1 | 6.70219 | 7.03619 | 6.36869 | 6.99563 | 5.19E-10 |
| WDSOF1 | 6.98917 | 7.34783 | 7.05433 | 7.31783 | 5.80E-10 |
| GLCCI1 | 9.388 | 9.63862 | 9.55415 | 9.78292 | 6.33E-10 |
| RAD1 | 6.13775 | 6.83075 | 6.07475 | 6.69775 | 7.89E-10 |
| LOC402521 | 8.64835 | 8.78417 | 8.18696 | 8.90391 | 8.78E-10 |
| THRAP2 | 7.70043 | 8.25919 | 7.4374 | 8.0594 | 8.96E-10 |
| KLHL9 | 7.51356 | 7.83311 | 7.12311 | 7.66533 | 9.28E-10 |
| C1orf22 | 7.17876 | 7.56786 | 6.44379 | 7.51821 | 9.76E-10 |
| KIF13B | 7.16829 | 7.35927 | 7.0602 | 7.4529 | 9.89E-10 |
| BCS1L | 6.5276 | 6.71067 | 6.2588 | 6.58147 | 1.24E-09 |
| UBP1 | 7.93888 | 8.1956 | 7.6152 | 8.01304 | 1.40E-09 |
| LMNB1 | 8.42907 | 8.75293 | 8.28813 | 8.84453 | 1.42E-09 |
| PIK3R2 | 6.83892 | 6.90317 | 6.815 | 6.8815 | 1.87E-09 |
| SCC-112 | 8.16405 | 8.32078 | 7.57 | 8.20966 | 2.83E-09 |
| HRMT1L3 | 6.64547 | 7.22947 | 6.47779 | 6.94526 | 2.97E-09 |
| ADAM10 | 8.56392 | 8.75416 | 8.22152 | 8.69184 | 3.87E-09 |
| DATF1 | 6.75127 | 7.00073 | 6.84236 | 7.07236 | 3.87E-09 |
| LYCAT | 6.89053 | 7.2116 | 6.45533 | 6.9792 | 4.61E-09 |
| CGI-119 | 8.0568 | 8.649 | 7.929 | 8.4472 | 4.62E-09 |
| GTF3C1 | 7.9023 | 8.09115 | 7.75094 | 8.07749 | 4.93E-09 |
| CDCA2 | 6.44257 | 7.08957 | 6.04764 | 6.93986 | 4.93E-09 |
| CTSO | 6.561 | 6.93257 | 6.13 | 6.97971 | 5.83E-09 |
| RPN2 | 9.2455 | 9.7184 | 9.3851 | 9.6503 | 6.12E-09 |
| WDR33 | 7.49093 | 7.71186 | 7.30286 | 7.69921 | 6.34E-09 |
| PPIL2 | 6.45838 | 6.86192 | 6.528 | 6.789 | 6.56E-09 |
| ZNF258 | 7.73278 | 7.73706 | 7.41283 | 7.6695 | 6.62E-09 |
| ALDH16A1 | 7.1621 | 7.44533 | 7.25829 | 7.41752 | 7.06E-09 |
| M11S1 | 10.3763 | 10.6446 | 10.3632 | 10.5784 | 7.52E-09 |
| TBC1D1 | 7.95087 | 8.21453 | 7.6492 | 8.04253 | 7.84E-09 |
| ATXN1 | 6.99533 | 7.10075 | 7.06117 | 7.388 | 7.84E-09 |
| PRKAG2 | 5.30587 | 5.34125 | 5.11525 | 5.468 | 8.80E-09 |
| FBXO30 | 7.8945 | 8.04175 | 7.35425 | 8.15875 | 9.25E-09 |
| MDM2 | 9.19343 | 9.50143 | 9.23986 | 9.55229 | 9.33E-09 |
| ALDH5A1 | 6.54544 | 6.94878 | 6.12589 | 6.81567 | 9.36E-09 |
| SHCBP1 | 7.9832 | 8.68587 | 7.70787 | 8.6296 | 9.76E-09 |
| H2AFX | 7.78133 | 8.39033 | 8.273 | 8.34933 | 9.98E-09 |
| TMEM48 | 7.43583 | 8.25539 | 7.024 | 7.80243 | 1.07E-08 |
| CDW92 | 7.68045 | 7.96409 | 7.52809 | 8.18364 | 1.31E-08 |
| SCARB2 | 7.33244 | 7.89344 | 7.015 | 7.86044 | 1.33E-08 |
| GLYBP | 6.97813 | 7.04644 | 6.61 | 6.95369 | 1.47E-08 |
| TRIM24 | 7.50408 | 7.76492 | 7.34585 | 7.66885 | 1.51E-08 |
| USP50 | 2.913 | 3.4106 | 3.2218 | 3.305 | 1.53E-08 |
| RAD23B | 7.90467 | 8.136 | 7.845 | 8.16317 | 1.55E-08 |
| RUNX1 | 7.43344 | 7.74776 | 7.5124 | 7.6568 | 1.67E-08 |
| DONSON | 6.90329 | 7.31986 | 6.97143 | 7.006 | 1.69E-08 |
| DERP6 | 8.0741 | 8.3322 | 8.1998 | 8.2357 | 1.70E-08 |
| C13orf18 | 8.42323 | 8.67277 | 7.84008 | 8.30777 | 1.70E-08 |
| AGPAT5 | 8.7003 | 8.7874 | 7.6872 | 8.48 | 1.79E-08 |
| DDOST | 9.77175 | 9.9155 | 9.73438 | 9.94462 | 1.99E-08 |
| SMARCA2 | 9.22819 | 9.59898 | 8.73256 | 9.30186 | 2.03E-08 |
| CCNA2 | 8.2388 | 8.9538 | 8.126 | 8.7492 | 2.22E-08 |
| PLXNC1 | 7.63519 | 7.82706 | 7.37038 | 7.69625 | 2.38E-08 |
| MIB1 | 7.85146 | 8.02523 | 7.50069 | 8.07123 | 3.11E-08 |
| ALMS1 | 5.89046 | 6.25246 | 5.60846 | 6.19831 | 3.29E-08 |
| TOP1 | 8.76971 | 9.14971 | 8.66643 | 9.15714 | 3.62E-08 |
| LANCL2 | 7.34246 | 8.02508 | 7.21738 | 8.06246 | 4.02E-08 |
| NS3TP1 | 7.09947 | 7.29307 | 6.76587 | 7.20533 | 4.70E-08 |
| SMC5L1 | 7.44208 | 7.6545 | 6.92575 | 7.51608 | 5.22E-08 |
| SLC30A1 | 7.28233 | 7.93933 | 7.17433 | 7.965 | 5.85E-08 |
| WSB1 | 9.29494 | 8.854 | 8.57894 | 8.58953 | 6.42E-08 |
| EXOC7 | 6.4995 | 6.79843 | 6.584 | 6.88714 | 6.51E-08 |
| SLC25A10 | 6.89892 | 7.40015 | 7.16231 | 7.24338 | 6.74E-08 |
| C10orf137 | 6.79938 | 7.30455 | 6.78421 | 7.31228 | 7.02E-08 |
| MBNL3 | 7.38143 | 7.87557 | 7.08314 | 7.60814 | 8.73E-08 |
| SIMP | 10.4279 | 10.4811 | 9.8217 | 10.3707 | 9.53E-08 |
| C1orf73 | 7.31104 | 7.75978 | 6.64859 | 7.54807 | 9.89E-08 |
| TXNL1 | 8.29618 | 8.61127 | 8.53091 | 8.52018 | 1.07E-07 |
| SPEN | 7.516 | 7.62771 | 7.20211 | 7.64046 | 1.36E-07 |
| SLBP | 8.24422 | 8.56133 | 8.14978 | 8.27356 | 1.37E-07 |
| ANXA11 | 9.796 | 9.80333 | 10.1596 | 9.9304 | 1.38E-07 |
| WDR77 | 8.16329 | 8.77014 | 8.224 | 8.71443 | 1.39E-07 |
| NUDT21 | 10.0264 | 10.4585 | 9.78727 | 10.2131 | 1.47E-07 |
| ZBTB40 | 6.29273 | 6.32521 | 5.77376 | 6.22249 | 1.51E-07 |
| CRR9 | 8.59 | 9.13459 | 8.61365 | 9.17753 | 1.51E-07 |
| TOP2A | 7.98249 | 8.87811 | 7.62881 | 8.62924 | 1.60E-07 |
| PCTK2 | 7.51516 | 7.77968 | 7.26021 | 7.62842 | 1.62E-07 |
| BAZ1A | 7.17805 | 7.4761 | 6.86114 | 7.25048 | 1.64E-07 |
| MCM7 | 8.58608 | 9.09464 | 8.3692 | 8.94136 | 1.71E-07 |
| AGPAT3 | 8.719 | 8.89444 | 8.71011 | 8.867 | 1.81E-07 |
| CASP4 | 7.33164 | 7.64218 | 7.59364 | 7.49382 | 1.89E-07 |
| CAS1 | 6.8493 | 7.23939 | 6.15565 | 7.13157 | 1.90E-07 |
| E2F7 | 7.16456 | 7.70667 | 7.10556 | 7.66789 | 1.92E-07 |
| CSRP2BP | 6.20389 | 6.69989 | 6.06444 | 6.48744 | 2.08E-07 |
| MAP3K5 | 8.77918 | 8.92059 | 8.25235 | 8.88724 | 2.41E-07 |
| PPRC1 | 6.95667 | 7.46933 | 7.03858 | 7.34867 | 2.43E-07 |
| ELL2 | 9.18767 | 9.43667 | 8.80633 | 9.403 | 2.46E-07 |
| CHAF1B | 6.45 | 6.89046 | 6.22708 | 6.72923 | 2.59E-07 |
| ANLN | 5.89679 | 6.65764 | 5.61064 | 6.39971 | 2.63E-07 |
| THADA | 6.64076 | 6.93293 | 6.17964 | 6.94511 | 2.70E-07 |
| VCPIP1 | 6.93971 | 7.18314 | 6.64514 | 7.11629 | 2.74E-07 |
| NUP214 | 7.989 | 8.32033 | 7.91567 | 8.15133 | 2.80E-07 |
| STS | 6.83418 | 7.577 | 6.95827 | 7.39055 | 2.83E-07 |
| TBCD | 8.02929 | 8.31876 | 7.99057 | 8.15443 | 2.88E-07 |
| CD44 | 9.33591 | 9.48771 | 9.36829 | 9.48819 | 3.02E-07 |
| BRF1 | 6.29829 | 6.41136 | 6.24921 | 6.39229 | 3.33E-07 |
| CCNL1 | 8.45336 | 8.35727 | 7.92582 | 8.25627 | 3.43E-07 |
| LOC116143 | 7.46536 | 7.55973 | 7.17555 | 7.60445 | 3.44E-07 |
| SLC4A5 | 7.61156 | 7.65894 | 7.32056 | 7.69675 | 3.55E-07 |
| ZNF289 | 7.89173 | 8.06413 | 7.88747 | 8.1128 | 3.79E-07 |
| HIPK2 | 8.70695 | 8.7761 | 8.50916 | 8.67684 | 3.88E-07 |
| PCTP | 6.84615 | 7.25138 | 6.98369 | 7.22738 | 4.06E-07 |
| DYM | 8.2152 | 8.3371 | 7.9847 | 8.0616 | 4.27E-07 |
| NKTR | 7.1736 | 7.28744 | 6.50544 | 7.17912 | 4.41E-07 |
| FBXO33 | 7.44186 | 7.812 | 7.27657 | 7.67586 | 4.72E-07 |
| APRIN | 6.77347 | 7.18213 | 6.25898 | 7.03902 | 4.76E-07 |
| BAG1 | 7.816 | 8.07015 | 8.11785 | 8.09692 | 5.14E-07 |
| HIC1 | 7.5025 | 7.815 | 7.529 | 7.75475 | 5.44E-07 |
| C14orf101 | 6.91667 | 7.23844 | 6.74289 | 7.18144 | 5.76E-07 |
| DEPDC1B | 7.17077 | 7.836 | 7.09462 | 7.63031 | 6.13E-07 |
| C3orf23 | 6.49078 | 6.79122 | 5.844 | 6.51667 | 6.16E-07 |
| ATAD2 | 6.6543 | 7.33552 | 6.16745 | 6.92879 | 6.16E-07 |
| UBE4B | 8.17139 | 8.23711 | 7.81322 | 8.18433 | 6.26E-07 |
| FOXM1 | 7.10753 | 7.89035 | 7.16282 | 7.58424 | 6.30E-07 |
| PSMD1 | 8.90007 | 9.14786 | 8.46321 | 9.14921 | 6.30E-07 |
| MAGEF1 | 7.87733 | 7.987 | 7.893 | 8.17433 | 6.60E-07 |
| HIRA | 7.72237 | 8.01511 | 7.59615 | 7.90007 | 6.66E-07 |
| BTAF1 | 8.2727 | 8.30405 | 7.7731 | 8.1518 | 6.74E-07 |
| LARS | 9.02509 | 8.96927 | 8.31673 | 8.85491 | 7.05E-07 |
| ECT2 | 6.36252 | 6.96348 | 5.80296 | 6.61793 | 7.10E-07 |
| AQR | 7.66083 | 8.02694 | 7.23944 | 7.85872 | 7.19E-07 |
| ZNF318 | 8.85135 | 9.00613 | 8.33606 | 8.62239 | 7.22E-07 |
| TYMS | 9.142 | 9.7668 | 9.24 | 9.6456 | 7.28E-07 |
| CASC5 | 6.00747 | 7.06218 | 5.93812 | 6.97065 | 7.34E-07 |
| DDX46 | 6.6105 | 7.055 | 5.98479 | 6.95043 | 7.41E-07 |
| TH1L | 8.55242 | 8.98979 | 8.71474 | 8.94453 | 7.46E-07 |
| HNRPDL | 8.3007 | 8.508 | 7.9292 | 8.3031 | 7.69E-07 |
| ADCY1 | 6.48106 | 6.88675 | 5.83075 | 6.2725 | 8.64E-07 |
| FAM82A | 4.19809 | 4.50245 | 4.06309 | 4.58309 | 8.89E-07 |
| LOC439991 | 9.53091 | 9.46255 | 9.42854 | 9.45273 | 8.93E-07 |
| DPYSL2 | 7.14167 | 7.30678 | 6.72922 | 7.31522 | 9.33E-07 |
| COCH | 5.02763 | 5.46325 | 5.388 | 5.682 | 9.43E-07 |
| SH3KBP1 | 10.045 | 10.1258 | 9.98848 | 10.0935 | 9.46E-07 |
| GTSE1 | 6.46147 | 7.28326 | 6.69179 | 6.96505 | 9.96E-07 |
| SEC23IP | 8.6557 | 9 | 8.56711 | 8.91956 | 1.03E-06 |
| HRB | 9.832 | 9.87158 | 9.46621 | 9.79463 | 1.03E-06 |
| UBE3C | 7.79659 | 7.89929 | 7.38306 | 7.80606 | 1.05E-06 |
| MTMR10 | 6.62867 | 6.9898 | 6.40227 | 6.75867 | 1.07E-06 |
| TARSL1 | 7.38627 | 7.46627 | 7.24127 | 7.38973 | 1.12E-06 |
| RREB1 | 7.44952 | 7.40992 | 7.37944 | 7.53552 | 1.13E-06 |
| FADS1 | 7.54286 | 7.08986 | 7.57343 | 6.84314 | 1.18E-06 |
| SAMD9 | 6.9408 | 6.8604 | 6.37733 | 7.1256 | 1.18E-06 |
| RANBP2 | 8.204 | 8.39127 | 7.42136 | 8.29073 | 1.22E-06 |
| LOC441093 | 5.54375 | 6.45313 | 5.44275 | 6.18213 | 1.27E-06 |
| IDE | 8.01556 | 8.40015 | 7.54459 | 8.15807 | 1.29E-06 |
| TTL | 6.95157 | 7.24957 | 6.63529 | 7.08029 | 1.29E-06 |
| CLSPN | 5.10076 | 5.76145 | 4.73303 | 5.67152 | 1.29E-06 |
| LOC159090 | 6.86035 | 7.28859 | 6.70624 | 7.04835 | 1.32E-06 |
| FANCD2 | 6.031 | 6.68926 | 5.66684 | 6.35389 | 1.35E-06 |
| FSIP2 | 5.17811 | 5.57967 | 4.76194 | 5.398 | 1.37E-06 |
| B3GALNT2 | 7.06062 | 7.19308 | 6.78738 | 7.20062 | 1.43E-06 |
| CHST2 | 7.97853 | 8.08589 | 7.98442 | 8.27421 | 1.43E-06 |
| TTC12 | 5.97189 | 6.59937 | 5.97926 | 6.56137 | 1.51E-06 |
| CPSF2 | 8.82061 | 9.18383 | 8.64722 | 9.06861 | 1.56E-06 |
| RTN4 | 7.91069 | 7.98146 | 7.57885 | 7.797 | 1.59E-06 |
| STAG1 | 6.72126 | 7.13794 | 6.25423 | 7.05103 | 1.60E-06 |
| DPF1 | 6.27527 | 6.43036 | 6.28127 | 6.49018 | 1.61E-06 |
| PB1 | 8.05319 | 8.2955 | 7.50394 | 8.28594 | 1.65E-06 |
| CYP20A1 | 5.87082 | 6.52294 | 5.71506 | 6.43471 | 1.67E-06 |
| DAZL | 4.8825 | 5.14825 | 4.81625 | 5.4135 | 1.72E-06 |
| C9orf40 | 6.85625 | 7.1875 | 6.96225 | 7.00825 | 1.79E-06 |
| APITD1 | 5.00889 | 5.22589 | 4.89144 | 5.22189 | 1.80E-06 |
| UBE2J1 | 9.07682 | 9.42553 | 8.92388 | 9.48223 | 1.81E-06 |
| FUBP1 | 9.35227 | 9.55627 | 8.93618 | 9.52718 | 1.84E-06 |
| TA-PP2C | 8.91133 | 8.88733 | 8.81933 | 8.8245 | 1.89E-06 |
| PIPOX | 5.9172 | 5.4246 | 5.682 | 5.4174 | 1.91E-06 |
| CLTC | 9.69215 | 9.97766 | 9.47517 | 9.98785 | 1.91E-06 |
| AMMECR1 | 7.38945 | 7.60145 | 7.33182 | 7.49309 | 1.96E-06 |
| PLCG2 | 8.93661 | 9.16085 | 8.76327 | 8.93915 | 1.98E-06 |
| EP300 | 8.02862 | 8.27604 | 7.77538 | 8.16209 | 2.07E-06 |
| TMEM30A | 8.53892 | 8.678 | 8.14169 | 8.74338 | 2.20E-06 |
| FLJ14681 | 8.29133 | 8.18833 | 7.57822 | 8.20811 | 2.22E-06 |
| KIAA1280 | 6.36595 | 6.62963 | 6.20505 | 6.39153 | 2.25E-06 |
| RINT-1 | 6.70236 | 6.83755 | 6.20591 | 6.74882 | 2.25E-06 |
| TMEM33 | 7.82636 | 8.01855 | 7.59909 | 7.98436 | 2.26E-06 |
| KIAA1961 | 7.60388 | 7.64433 | 7.05257 | 7.48527 | 2.38E-06 |
| GANAB | 8.81954 | 9.19777 | 8.68785 | 9.11592 | 2.39E-06 |
| FLJ20257 | 7.95123 | 8.14139 | 7.85477 | 8.21862 | 2.41E-06 |
| ZBTB4 | 6.848 | 6.91176 | 6.85612 | 6.85 | 2.43E-06 |
| PLRG1 | 9.91631 | 10.112 | 9.74815 | 10.1195 | 2.45E-06 |
| MAML1 | 7.71431 | 7.75692 | 7.75508 | 7.72677 | 2.51E-06 |
| C6orf167 | 6.235 | 6.63393 | 5.57733 | 6.3702 | 2.53E-06 |
| ZNF664 | 7.86046 | 8.06138 | 7.79831 | 7.81092 | 2.58E-06 |
| ANKRD13C | 7.7873 | 7.90574 | 7.24261 | 7.82174 | 2.58E-06 |
| TEF | 6.41067 | 6.60933 | 6.63378 | 6.64467 | 2.60E-06 |
| KLHL2 | 6.80108 | 6.91815 | 6.37215 | 6.97446 | 2.65E-06 |
| POMGNT1 | 6.94088 | 6.9944 | 6.7904 | 6.96296 | 2.66E-06 |
| FLJ43276 | 6.13114 | 6.718 | 6.17 | 6.54657 | 2.71E-06 |
| POLA2 | 7.74518 | 8.37936 | 7.82845 | 8.17691 | 2.78E-06 |
| NCOA7 | 7.04892 | 7.23277 | 6.25631 | 6.85615 | 2.81E-06 |
| SOD1 | 8.65109 | 9.14727 | 8.78164 | 8.99382 | 2.87E-06 |
| MGC13057 | 4.96967 | 5.342 | 5.23433 | 5.59233 | 2.97E-06 |
| LOC391613 | 8.12086 | 8.73143 | 8.24143 | 8.69743 | 2.98E-06 |
| ELAC2 | 8.18667 | 8.68333 | 8.18625 | 8.46892 | 3.00E-06 |
| THOC1 | 6.362 | 6.8796 | 5.9977 | 6.529 | 3.05E-06 |
| LOC144742 | 7.33922 | 7.68878 | 7.20522 | 7.63113 | 3.10E-06 |
| SLC20A2 | 6.97463 | 7.32042 | 6.86232 | 7.11716 | 3.14E-06 |
| LONP | 8.74922 | 8.82511 | 8.41844 | 8.70378 | 3.15E-06 |
| MYO1D | 8.00761 | 8.29368 | 7.30574 | 7.69116 | 3.16E-06 |
| DNMT1 | 7.75576 | 8.1341 | 7.71876 | 8.05538 | 3.24E-06 |
| TTLL4 | 7.29569 | 7.56123 | 7.02562 | 7.30208 | 3.26E-06 |
| C14orf93 | 6.41492 | 6.52369 | 6.25108 | 6.42785 | 3.27E-06 |
| STXBP5 | 6.61212 | 6.74848 | 5.96618 | 6.49249 | 3.28E-06 |
| MAPKAPK2 | 9.32229 | 9.59257 | 9.43457 | 9.61 | 3.41E-06 |
| XYLT1 | 7.4924 | 7.768 | 7.4716 | 7.6768 | 3.50E-06 |
| MPDU1 | 8.8134 | 9.4684 | 9.1518 | 9.4372 | 3.54E-06 |
| WDR56 | 5.95231 | 6.11569 | 5.32415 | 5.98969 | 3.62E-06 |
| FBXO4 | 7.50764 | 7.74636 | 7.46036 | 7.85527 | 3.70E-06 |
| L3MBTL2 | 7.33536 | 7.48309 | 7.29927 | 7.51327 | 3.72E-06 |
| CXorf21 | 6.0732 | 6.81 | 6.234 | 6.8088 | 3.78E-06 |
| ZXDC | 6.93943 | 7.14514 | 6.76971 | 7.09657 | 3.82E-06 |
| CDYL | 7.77843 | 7.73714 | 7.68271 | 7.82643 | 3.92E-06 |
| ACBD3 | 8.66463 | 8.6045 | 8.27925 | 8.61 | 3.99E-06 |
| LY75 | 9.61723 | 9.38804 | 8.81604 | 9.06196 | 4.00E-06 |
| DDX21 | 8.77391 | 9.20945 | 8.209 | 8.84345 | 4.28E-06 |
| COQ4 | 7.49183 | 7.72167 | 7.7615 | 7.70083 | 4.39E-06 |
| MPP5 | 6.04726 | 6.33895 | 5.77558 | 6.15547 | 4.40E-06 |
| GPI | 11.1187 | 11.2342 | 11.0942 | 10.86 | 4.41E-06 |
| FAM53C | 7.4915 | 7.686 | 7.2356 | 7.5608 | 4.46E-06 |
| BIVM | 5.84825 | 6.24325 | 5.40425 | 5.98775 | 4.56E-06 |
| ARFGEF1 | 7.3729 | 7.62971 | 6.97535 | 7.54249 | 4.60E-06 |
| GAK | 8.06747 | 7.99535 | 8.06971 | 8.10453 | 4.68E-06 |
| MAPKAPK5 | 7.65435 | 7.98118 | 7.70671 | 7.85059 | 4.82E-06 |
| FAM49B | 6.95463 | 7.61462 | 7.12425 | 7.41538 | 4.82E-06 |
| TJP2 | 7.90019 | 8.04535 | 7.57536 | 7.92781 | 4.92E-06 |
| FLJ10979 | 7.6199 | 7.6953 | 7.2771 | 7.7106 | 4.97E-06 |
| MYH3 | 7.69422 | 7.89 | 7.76689 | 7.83178 | 5.02E-06 |
| KIAA0090 | 7.87993 | 8.1411 | 7.55855 | 8.13476 | 5.05E-06 |
| ZNF367 | 6.59156 | 6.93733 | 6.59978 | 6.828 | 5.27E-06 |
| BRPF1 | 6.89571 | 7.10867 | 6.74971 | 7.10448 | 5.47E-06 |
| TP53INP2 | 6.7956 | 6.9492 | 6.8592 | 6.8286 | 5.85E-06 |
| C2orf30 | 7.8715 | 7.98987 | 7.59662 | 8.02475 | 5.89E-06 |
| MGC5242 | 8.7178 | 8.8354 | 8.613 | 8.8954 | 5.90E-06 |
| MTDH | 9.93188 | 10.1644 | 9.60918 | 10.1045 | 6.14E-06 |
| GATA3 | 6.54185 | 6.69231 | 6.59185 | 6.64138 | 6.46E-06 |
| MGC10850 | 5.5125 | 6.131 | 5.691 | 6.0175 | 6.60E-06 |
| RAD18 | 7.21176 | 7.46518 | 6.83129 | 7.334 | 6.65E-06 |
| RASSF5 | 9.00333 | 8.87633 | 8.92883 | 8.79417 | 6.72E-06 |
| RFK | 7.24267 | 7.52967 | 7.425 | 7.386 | 6.76E-06 |
| HNRPA0 | 9.54511 | 9.61044 | 9.38111 | 9.74844 | 7.25E-06 |
| PDXK | 8.47882 | 8.442 | 8.50415 | 8.47993 | 7.26E-06 |
| WDR36 | 8.48344 | 8.61416 | 7.77744 | 8.51152 | 7.68E-06 |
| PDHX | 7.78371 | 8.07714 | 7.68457 | 8.06386 | 7.69E-06 |
| ZYG11BL | 7.47181 | 7.57619 | 7.35333 | 7.43133 | 7.76E-06 |
| IARS2 | 8.65561 | 8.78026 | 8.15587 | 8.61045 | 7.94E-06 |
| C20orf129 | 6.76091 | 7.44873 | 6.74036 | 7.08436 | 8.23E-06 |
| ST7 | 8.73125 | 8.90992 | 8.40058 | 8.66442 | 8.29E-06 |
| USP52 | 6.34247 | 6.38935 | 6.139 | 6.30741 | 8.44E-06 |
| ATXN2L | 7.99539 | 8.07217 | 7.99496 | 7.88096 | 8.67E-06 |
| RER1 | 9.05 | 9.3156 | 9.0024 | 9.2706 | 9.05E-06 |
| ZNF592 | 7.71385 | 7.89754 | 7.83723 | 7.83923 | 9.10E-06 |
| MFNG | 8.6464 | 9.04 | 8.8484 | 8.7266 | 9.18E-06 |
| SMARCD1 | 8.44962 | 8.76476 | 8.41724 | 8.65552 | 9.18E-06 |
| CBS | 6.05326 | 5.63347 | 5.734 | 5.48232 | 9.38E-06 |
| SLC25A3 | 5.88844 | 6.22578 | 5.68756 | 5.86578 | 9.60E-06 |
| ABCB10 | 5.9344 | 6.231 | 5.69 | 6.1092 | 9.63E-06 |
| RFXDC2 | 7.48529 | 7.76771 | 7.06914 | 7.42971 | 9.76E-06 |
| CUL4A | 7.342 | 7.594 | 7.15442 | 7.56558 | 9.88E-06 |
| UBE1DC1 | 8.29035 | 8.39777 | 7.65718 | 8.37224 | 1.00E-05 |
| CES2 | 7.0312 | 7.1841 | 7.0428 | 7.202 | 1.01E-05 |
| LIPA | 8.85253 | 8.9456 | 8.61293 | 8.9148 | 1.02E-05 |
| CHKA | 7.24965 | 7.71012 | 7.13353 | 7.64894 | 1.02E-05 |
| DCTN4 | 8.72893 | 8.74507 | 8.47053 | 8.73293 | 1.03E-05 |
| WDR4 | 7.166 | 7.60727 | 7.13018 | 7.47745 | 1.03E-05 |
| EHD1 | 8.1904 | 8.23693 | 8.18413 | 8.19733 | 1.03E-05 |
| STIM2 | 6.7005 | 6.9192 | 6.3822 | 6.8416 | 1.04E-05 |
| ROCK2 | 6.52729 | 6.68259 | 5.57482 | 6.40106 | 1.05E-05 |
| NOL6 | 7.677 | 8.05025 | 7.66756 | 7.93587 | 1.05E-05 |
| ATP11B | 7.13343 | 7.144 | 6.67686 | 7.22971 | 1.06E-05 |
| UBR1 | 7.38649 | 7.71551 | 7.04973 | 7.70644 | 1.09E-05 |
| ADM | 7.6705 | 6.794 | 7.86325 | 6.999 | 1.13E-05 |
| MARK2 | 7.551 | 7.82873 | 7.59791 | 7.64373 | 1.15E-05 |
| C8orf35 | 6.55158 | 6.79516 | 6.08063 | 6.70074 | 1.16E-05 |
| AKT1S1 | 7.08818 | 7.30145 | 7.19309 | 7.15709 | 1.17E-05 |
| CNDP2 | 8.92422 | 9.15656 | 8.83567 | 9.15744 | 1.18E-05 |
| SLC39A3 | 6.8915 | 6.95175 | 6.9805 | 6.9405 | 1.20E-05 |
| TOLLIP | 7.73764 | 7.94891 | 7.86745 | 7.998 | 1.20E-05 |
| KLF5 | 6.61738 | 6.82077 | 6.66323 | 6.73108 | 1.20E-05 |
| ZNF406 | 6.9004 | 7.24016 | 6.80912 | 7.1392 | 1.23E-05 |
| TMEM55A | 7.03818 | 7.37109 | 7.09127 | 7.11491 | 1.25E-05 |
| BCAS3 | 6.30382 | 6.51164 | 6.38339 | 6.58879 | 1.25E-05 |
| KPNB1 | 9.92373 | 10.2425 | 9.86636 | 10.0857 | 1.26E-05 |
| ENTH | 7.974 | 8.08762 | 7.68175 | 8.03738 | 1.27E-05 |
| NUP98 | 8.0234 | 8.34072 | 7.97017 | 8.20911 | 1.29E-05 |
| RNF167 | 8.24973 | 8.436 | 8.47787 | 8.48107 | 1.32E-05 |
| FLJ20232 | 7.49917 | 7.72683 | 7.4085 | 7.65867 | 1.33E-05 |
| BZW2 | 4.63513 | 5.12337 | 4.43113 | 4.89025 | 1.35E-05 |
| FLJ42094 | 6.34923 | 6.44554 | 6.492 | 6.64754 | 1.38E-05 |
| HT008 | 6.88314 | 7.206 | 6.68305 | 7.13038 | 1.39E-05 |
| ZFP1 | 6.10156 | 6.50089 | 6.00133 | 6.41644 | 1.46E-05 |
| SIL | 6.29387 | 6.805 | 5.754 | 6.4624 | 1.46E-05 |
| SLC35F5 | 7.49038 | 7.4119 | 6.78257 | 7.35248 | 1.47E-05 |
| MTMR2 | 8.74067 | 8.9461 | 8.43019 | 8.79971 | 1.48E-05 |
| DKC1 | 8.13656 | 8.54122 | 8.10133 | 8.47778 | 1.49E-05 |
| PORCN | 7.09867 | 7.2516 | 7.03867 | 7.17133 | 1.50E-05 |
| CAMK2A | 4.85718 | 4.83576 | 4.90024 | 4.89059 | 1.51E-05 |

*Supplemental Table 5.*

There were 8 genes with statistically significant Diagnosis x Glucose deprivation x Probeset interaction effects on expression after Bonferroni correction. The gene symbol, mean expression level and p-value are shown in the table below. ( C – Control; SZ – Schizophrenia; GD – Glucose Deprivation; NG – Normal Glucose; FC – Fold Change).

| **Gene Symbol** | **Mean (C+GD)** | **Mean (C+NG)** | **Mean**  **(SZ+GD)** | **Mean**  **(SZ+NG)** | **p-value (Diagnosis x Glucose deprivation x Probeset)** |
| --- | --- | --- | --- | --- | --- |
| FBXW5 | 7.88647 | 8.03106 | 8.12341 | 7.99412 | 1.39E-12 |
| PPARGC1B | 7.32869 | 7.17669 | 7.21692 | 7.257 | 7.10E-09 |
| PPP4R1 | 7.89607 | 8.18821 | 7.70752 | 8.08159 | 2.78E-07 |
| GLS | 8.82062 | 8.94821 | 8.31662 | 9.06 | 3.81E-07 |
| KIAA1404 | 8.97911 | 8.82633 | 8.745 | 9.02144 | 3.83E-07 |
| RAC3 | 6.31657 | 6.49286 | 6.49286 | 6.15143 | 3.25E-06 |
| PHC3 | 9.03095 | 9.05568 | 8.80158 | 9.00284 | 5.63E-06 |
| NPHP3 | 6.28087 | 6.24974 | 5.66096 | 5.89591 | 1.27E-05 |

Supplemental Table 6. The results of this study were compared with a recent study of microarray gene expression in the dorsolateral cortex of subjects with schizophrenia and control subjects. Of the 54 genes identified by Maycox et al (2009) [1], there were 12 genes with diagnosis or Diagnosis x Probeset effects displaying nominal p-values < 0.05 in the current study The gene symbol, means and p-values are shown in the table below. (C – Control; SZ – Schizophrenia; GD – Glucose Deprivation; NG – Normal Glucose; FC – Fold Change).

| **Gene Symbol** | **p-value**  **(Diagnosis x Probeset)** | **p-value**  **(Diagnosis)** | **p-value**  **(Diagnosis x**  **Glucose x**  **Probeset)** | **Mean**  **(C +NG)** | **Mean**  **(C + GD)** | **Mean**  **(Sz + NG)** | **Mean**  **(Sz + GD)** |
| --- | --- | --- | --- | --- | --- | --- | --- |
| WNK1 | 0.00 | 0.15 | 0.94 | 8.45 | 8.59 | 8.16 | 8.41 |
| JMJD1A | 0.00 | 0.07 | 0.89 | 8.61 | 8.42 | 7.83 | 7.95 |
| PHYHIP | 0.00 | 0.29 | 0.83 | 5.16 | 5.29 | 5.36 | 5.28 |
| NEK7 | 0.01 | 0.01 | 0.38 | 8.57 | 8.42 | 7.95 | 8.15 |
| UQCRC1 | 0.01 | 0.54 | 0.62 | 8.36 | 8.41 | 8.50 | 8.39 |
| ARPP-19 | 0.02 | 0.06 | 0.96 | 9.70 | 9.89 | 9.39 | 9.74 |
| ZNF395 | 0.04 | 0.46 | 0.48 | 5.28 | 5.73 | 5.18 | 5.22 |
| NAP1L1 | 0.05 | 0.13 | 0.11 | 8.15 | 8.76 | 7.41 | 8.23 |
| OLFM1 | 0.05 | 0.52 | 0.26 | 4.30 | 4.32 | 4.46 | 4.29 |
| NEK7 | 0.01 | 0.01 | 0.38 | 8.57 | 8.42 | 7.95 | 8.15 |
| RCBTB1 | 0.16 | 0.03 | 0.59 | 6.78 | 6.98 | 6.12 | 6.72 |
| GNG3 | 0.98 | 0.03 | 0.87 | 4.74 | 4.75 | 5.04 | 4.88 |
