# Supplementary material for: Exon expression in lymphoblastoid cell lines from subjects with schizophrenia before and after glucose deprivation
Source: BMC Med Genomics. 2009 Sep 22;2:62. doi: 10.1186/1755-8794-2-62 (PMC2760574; doi:10.1186/1755-8794-2-62)
Supplement: Additional File 3 — PPARGC1B and FBXW5 expression. Figures displaying significant Diagnosis × Glucose deprivation × Probeset interaction effects on transcripts expression for genes PPARGC1B and FBXW5. [file 1755-8794-2-62-S3.DOC]

*Supplementary Figures 1-2.* Significant Diagnosis x Glucose deprivation x Probeset interaction effect on transcripts expression. For each gene, the top graph depicts transcript expression by exon during glucose deprivation and the bottom graph depicts transcript expression by exon under normal glucose conditions. The probeset number is shown on the x-axis, and the average group expression on the y-axis.

Supplementary Figure 1. A.) There was a significant Diagnosis x Glucose deprivation x Probeset interaction effect on FBXW5 expression. B.) The probeset with the most significant diagnosis x glucose interaction was probeset 3230531 (p = 0.007) and is depicted by dot plot. In control subjects there was a significant increase in probeset expression following glucose deprivation (p = 0.002). In contrast, in subjects with schizophrenia there was a trend toward a decrease in probeset expression following glucose deprivation (p = 0.08).

A.)

*
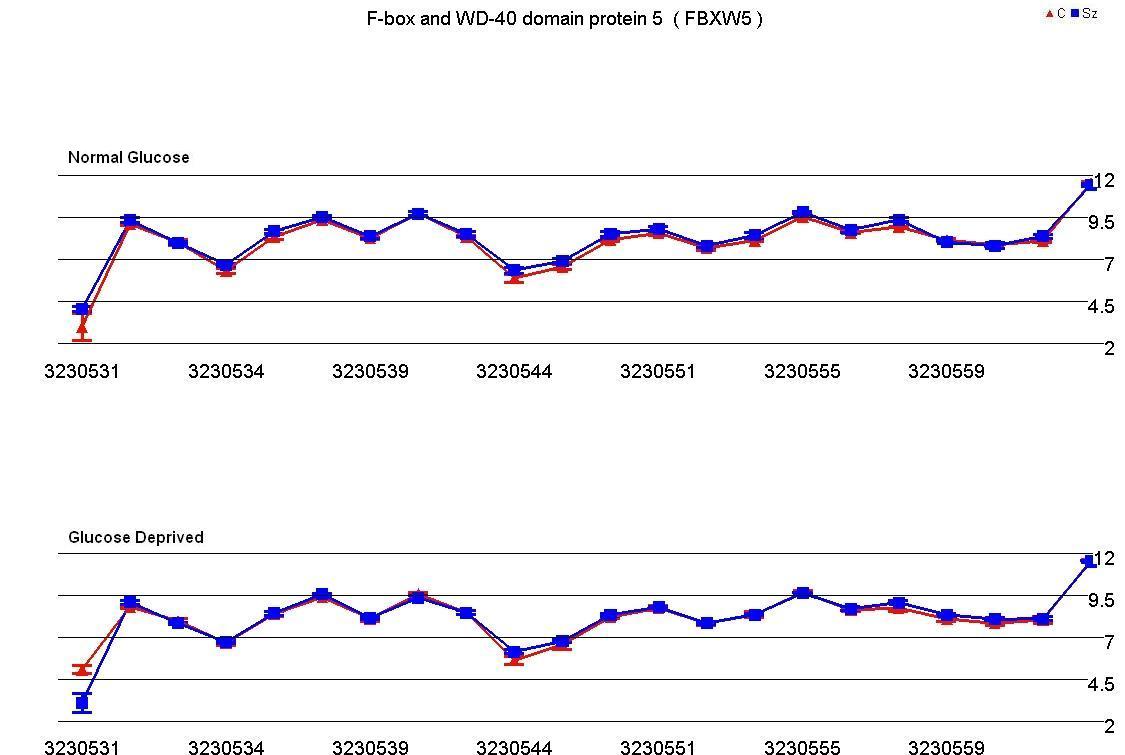
*

B.)

*
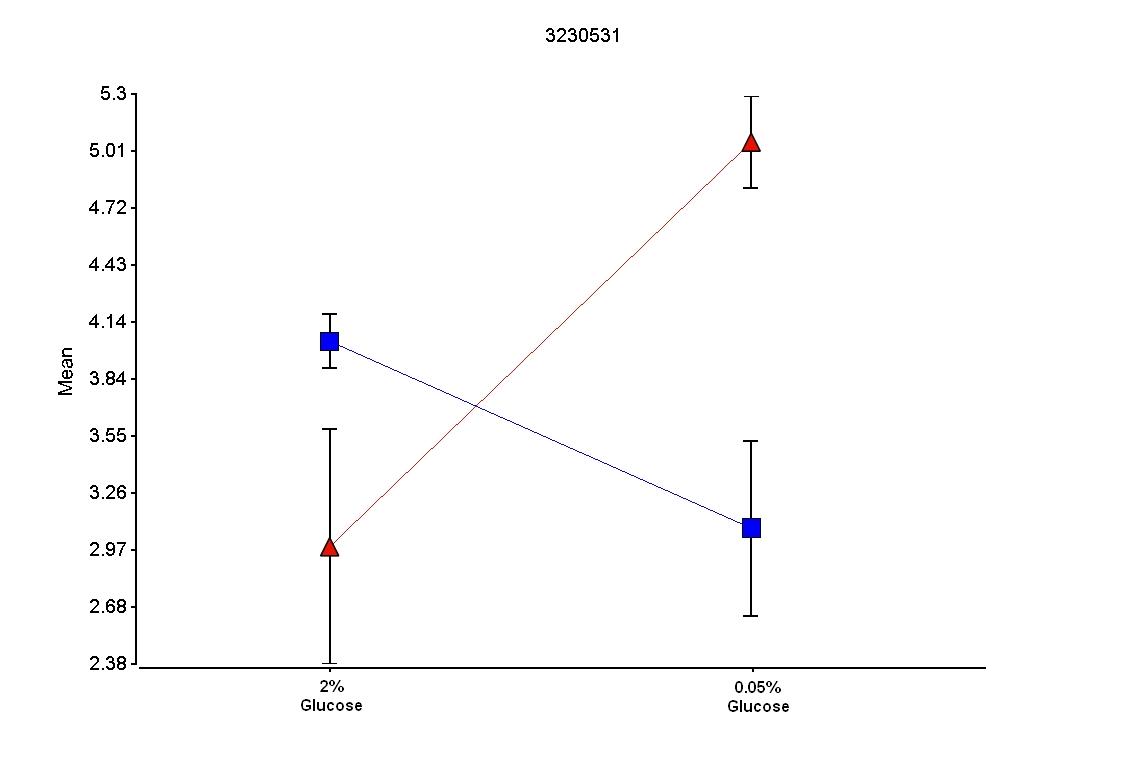
*

Supplementary Figure 2. A.) There was a significant Diagnosis x Glucose deprivation x Probeset interaction effect on PPARGC1B expression. B.) The probeset with the most significant diagnosis x glucose interaction was probeset 2835214 (p = 0.006). Posthoc analysis revealed a significant increase in probeset expression in subjects with schizophrenia following glucose deprivation (p = 0.02).

A.)


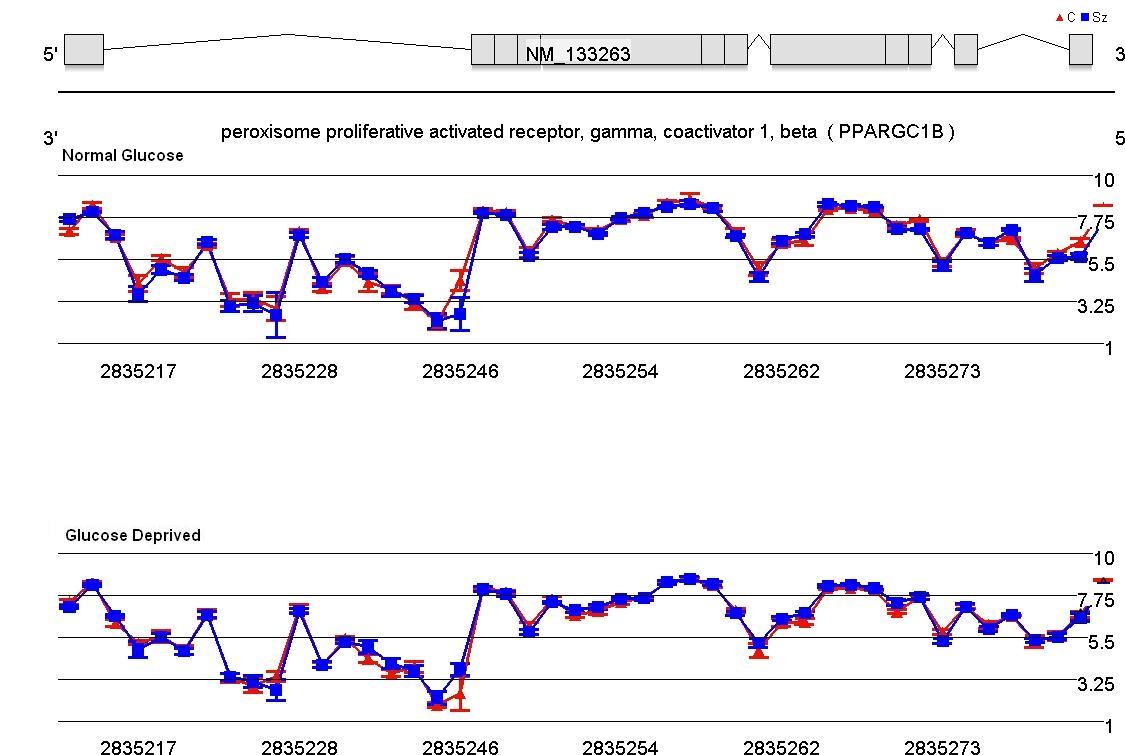


B.)

*
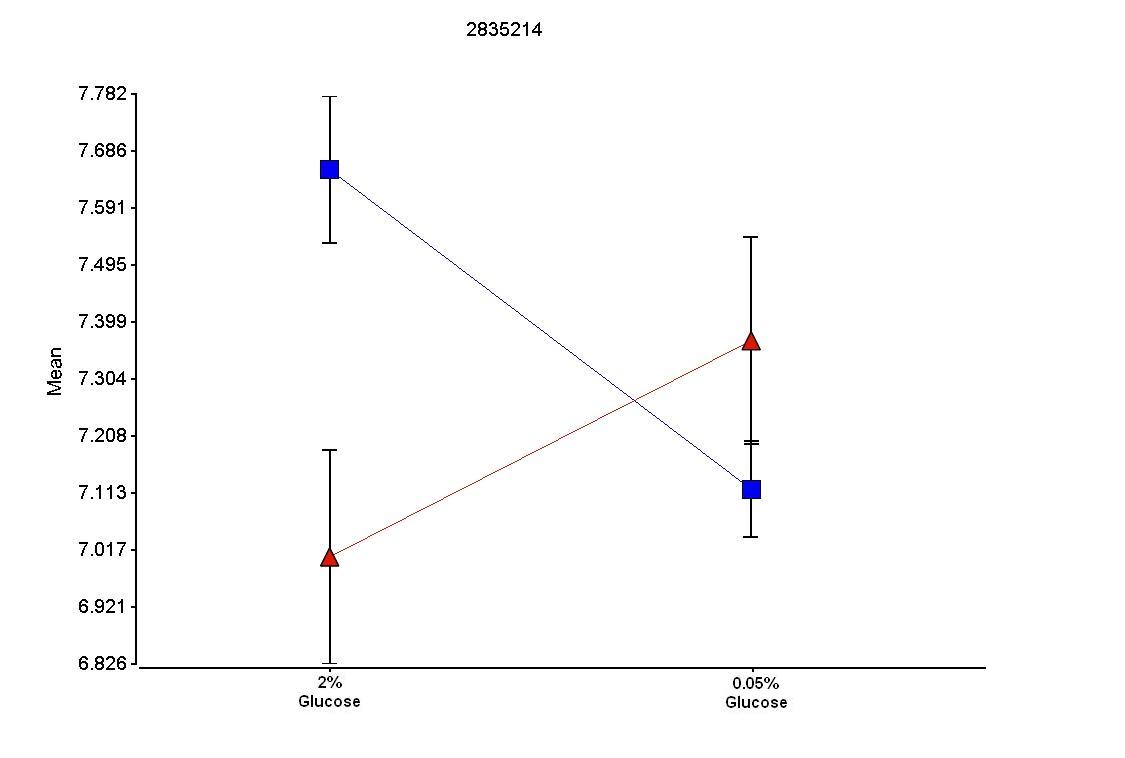
*

1. Maycox PR, Kelly F, Taylor A, Bates S, Reid J, Logendra R, Barnes MR, Larminie C, Jones N, Lennon M et al**: Analysis of gene expression in two large schizophrenia cohorts identifies multiple changes associated with nerve terminal functi**on. Mol Psychiatry 2009.
